# Rapid Genome Evolution and Adaptation of *Thlaspi arvense* Mediated by Recurrent RNA-Based and Tandem Gene Duplications

**DOI:** 10.3389/fpls.2021.772655

**Published:** 2022-01-04

**Authors:** Yanting Hu, Xiaopei Wu, Guihua Jin, Junchu Peng, Rong Leng, Ling Li, Daping Gui, Chuanzhu Fan, Chengjun Zhang

**Affiliations:** ^1^Germplasm Bank of Wild Species, Kunming Institute of Botany, Chinese Academy of Sciences, Kunming, China; ^2^University of Chinese Academy of Sciences, Beijing, China; ^3^Key Laboratory for Plant Diversity and Biogeography of East Asia, Kunming Institute of Botany, Chinese Academy of Sciences, Kunming, China; ^4^Department of Biological Sciences, Wayne State University, Detroit, MI, United States; ^5^Haiyan Engineering & Technology Center, Kunming Institute of Botany, Chinese Academy of Sciences, Kunming, China

**Keywords:** *Thlaspi arvense*, LTR retrotransposons, retroduplication, tandem duplication, genome adaptation, gene family

## Abstract

Retrotransposons are the most abundant group of transposable elements (TEs) in plants, providing an extraordinarily versatile source of genetic variation. *Thlaspi arvense*, a close relative of the model plant *Arabidopsis thaliana* with worldwide distribution, thrives from sea level to above 4,000 m elevation in the Qinghai-Tibet Plateau (QTP), China. Its strong adaptability renders it an ideal model system for studying plant adaptation in extreme environments. However, how the retrotransposons affect the *T. arvense* genome evolution and adaptation is largely unknown. We report a high-quality chromosome-scale genome assembly of *T. arvense* with a scaffold N50 of 59.10 Mb. Long terminal repeat retrotransposons (LTR-RTs) account for 56.94% of the genome assembly, and the *Gypsy* superfamily is the most abundant TEs. The amplification of LTR-RTs in the last six million years primarily contributed to the genome size expansion in *T. arvense.* We identified 351 retrogenes and 303 genes flanked by LTRs, respectively. A comparative analysis showed that orthogroups containing those retrogenes and genes flanked by LTRs have a higher percentage of significantly expanded orthogroups (SEOs), and these SEOs possess more recent tandem duplicated genes. All present results indicate that RNA-based gene duplication (retroduplication) accelerated the subsequent tandem duplication of homologous genes resulting in family expansions, and these expanded gene families were implicated in plant growth, development, and stress responses, which were one of the pivotal factors for *T. arvense*’s adaptation to the harsh environment in the QTP regions. In conclusion, the high-quality assembly of the *T. arvense* genome provides insights into the retroduplication mediated mechanism of plant adaptation to extreme environments.

## Introduction

*Thlaspi arvense* is a member of the extended II lineage of Brassicaceae (Huang et al., 2016), which is closely related to *Arabidopsis*. *T. arvense* is native to Eurasia and has a wide distribution in various temperate regions of the northern hemisphere (Warwick et al., 2002). *T. arvense* plants can survive in a wide altitude distribution from 0 to 4,000 m in the Qinghai-Tibet Plateau (QTP) region, the world’s highest plateau as a consequence of the continuous rising from the late Tertiary/mid-Miocene to the Quaternary, thus producing extensive genetic divergence and great species diversity (An et al., 2015; Zhang et al., 2019). Haplotypes of *T. arvense* unique to the QTP were recently recognized and collected. Based on a phylogeographic analysis, populations of *T. arvense* in China are a mixture of highly diverged ancestral subpopulations (An et al., 2015). The broad biodistribution of the Chinese *T. arvense* population demonstrates that *T. arvense* has relatively strong adaptability to environmental changes. Furthermore, *T. arvense* has been well recognized as a potential winter cover biofuel crop given its extreme cold tolerance and high seed oil content (Dorn et al., 2013, 2015; Sedbrook et al., 2014; Claver et al., 2017).

Transposable elements (TEs) are one of the major driving forces in genome and gene evolution (Fedoroff, 2012; Elbarbary et al., 2016). There are two main classes of TEs: Retrotransposons (class I), which transpose *via* an RNA intermediate with a “copy-and-paste” mechanism, and DNA transposons (class II), which transpose without an RNA intermediate through a “cut-and-paste” mechanism (Wicker et al., 2007). Among the class I, long terminal repeat retrotransposons (LTR-RTs) are the most abundant component in the genome of flowering plants and provide an extensive source of mutations and genetic variations (Feschotte et al., 2002; Lisch, 2012). LTR-RTs can contribute to the formation of retrogenes by retrotransposition, where a gene’s messenger RNA (mRNA) is captured, reverse transcribed, and integrated into new genomic positions by a retrotransposon (Kaessmann et al., 2009). The most remarkable feature of these RNA-based duplicated genes is the lack of introns compared with their parental genes. Compared with retroduplication driven by non-LTR retrotransposon (LINE/SINE), retrogenes mediated by LTR-RTs are flanked by LTR sequences which can function as donors of promoter regions for a novel gene copy (Casola and Betrán, 2017) and are prone to ectopic recombination (Stritt et al., 2020). LTR-RTs flanking genes in plants were first reported in maize (Jin and Bennetzen, 1994), where the *Bs1* LTR-RT transduced sequences from three different host genes. Further study showed its specific expression in reproductive tissues, e.g., post-pollen tassel (Elrouby and Bureau, 2010). In tomatoes, the *SUN* gene mediated by *Rider* LTR-RT led to an elongated fruit shape (Xiao et al., 2008). The genome-wide identification of LTR-RT-mediated retrogenes has been reported in plants as well as animals. For example, 27 retrogenes within LTR-RTs in the rice genome were identified, and the analysis demonstrated that the mechanism of the formation of free retrogenes is different from that of retrogenes inside LTR-RTs (Wang et al., 2006). Additionally, three retrogenes within LTR-RTs based on *Arabidopsis* resequencing data were recently reported (Zhu et al., 2016). A specific expression of 10 polymorphic retrogenes flanked by LTR-RTs in fruit flies and mice were detected in the testis, ovary, and head (Tan et al., 2016). A recent genome-wide survey in hot peppers reported numerous nucleotide-binding and leucine-rich-repeat (NLR) disease-resistance genes located inside LTR-RTs (105, 123, and 86 in *Capsicum annuum*, *Capsicum baccatum*, and *Capsicum chinense*, respectively). Some of these genes were set as being retroduplicated by LTR-RTs even when the parental genes were not identified (Kim et al., 2017). This study in peppers concluded that retroduplications played key roles in the massive emergence of disease-resistance genes (Kim et al., 2017). However, how retrotransposons, especially LTR-RTs, facilitate genome evolution and organismal adaptation has seldomly been explored.

The combination of single-molecule long-read sequencing, optical mapping, and chromosome conformation capture technologies have greatly improved the assembly of plant genomes, which contain a high proportion of repetitive sequences (Zhang et al., 2018; Hu et al., 2019; Wang et al., 2020). The genome size of *T. arvense* (2*n* = 14) is approximately 539 Mb (Johnston et al., 2005). An next-generation sequencing (NGS)-based assembly (343 Mb) of the draft *T. arvense* genome only accounted for 63.6% of the predicted genome size, with a great discontinuity and large gaps that hindered its utility for more accurate genome studies (Dorn et al., 2015). To better understand the genome evolution and expansion and further unravel the broad adaptability of *T. arvense* to environmental changes, we performed a *de novo* assembly of the *T. arvense* genome using a combination of single-molecule real-time (SMRT) sequencing (PacBio) (Berlin et al., 2015; Jiao et al., 2017), optical mapping (BioNano) (Jiao et al., 2017), and chromosome conformation capture (Hi-C) technologies (Mascher et al., 2017). Comparable to the newly published complete *T. arvense* genome (Geng et al., 2021), we present an additional genome assembly with a high level of continuity and annotation. The genomic analysis identified recent LTR-RT amplification events between 0 and 6 million years ago (MYA), which was responsible for the *T. arvense* genome size expansion. Comparative analyses of gene families between *T. arvense* and other three relative species in Brassicaceae suggest that LTR-RT amplification during the Pleistocene and late Pliocene contributed to the adaptation of *T. arvense* to the glacial-interglacial cycle in the QTP region. Gene family expansions mediated by retroduplication, especially LTR-RT mediated retroduplication, and the subsequent tandem duplication of homologous genes provided the caliper of development and stress resistance of *T. arvense* to cope with the harsh environment.

## Results

### Genome Assembly, Assessment, and Annotation

**FIGURE 1:**
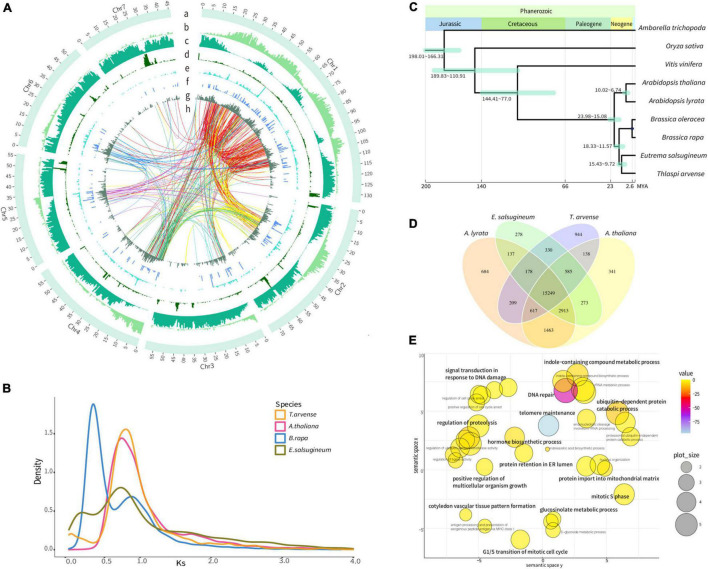
Genome features and evolutionary analyses of the *Thlaspi arvense* genome. **(A)** Distribution of genomic features [Track a, seven chromosomes. One scale label indicates 1 Mb. Track b, gene density indicated by gene length/500 Kb. Track c, *Gypsy* LTR-RTs density, indicated by *Gypsy* length/500 Kb. Track d, *Copia* LTR-RTs density, indicated by *Copia* length/500 Kb. Track e, LINE density, indicated by LINE length/500 Kb. Track f, SINE density, indicated by SINE length/500 Kb. Track g, DNA transposons density, indicated by DNA transposons length/500 Kb. Region h, transpositions tracks of the retrogene from its parental gene in Chr 1–7 are colored in red, yellow, light blue, green, purple, blue, and light purple lines, respectively]. **(B)** Density plot of the rate of synonymous distribution (Ks) values of the best reciprocal BLASTP hits in the genomes of *T. arvense*, *Eutrema salsugineum*, *Brassica rapa*, and *Arabidopsis thaliana*. **(C)** The phylogenetic tree of *T. arvense* with eight other angiosperms. **(D)** Venn diagram showing unique and shared orthogroups between genomes of *T. arvense* and three Brassicaceae close relatives. **(E)** REViGO semantic similarity scatterplot of the GO enrichment of significantly expanded orthogroups in *T. arvense* [bubble color indicates the provided *p*-value; bubble size indicates the frequency of the GO term in the underlying database (bubbles of more general terms are larger)].

We sequenced and collected 23.5 Gb Illumina HiSeq data for the genome survey. The estimated genome size, heterozygosity rate, and repeats rate are 487.20 Mb, 0.04%, and 41.73%, respectively (Supplementary Table 1 and Supplementary Figure 1). We generated 27.99 Gb (∼ 52×) subread sequences of *T. arvense* genome using SMRT sequencing technology from the PacBio Sequel platform (PacBio, 1305 O’Brien Dr., Menlo Park, CA, United States) with an average read length of 8.9 Kb (Supplementary Figure 2). We also collected 4.6 Gb optical map sequencing data from the BioNano Genomics Saphyr platform and 32.3 Gb Hi-C high-throughput chromosome conformation capture sequencing data. By combining all these data, we assembled a chromosome-scale *T. arvense* genome by conducting contigs assembly, scaffolds assembly, pseudochromosome construction, as well as rounds of polishing and gap filling (Supplementary Table 2). The final assembled genome size was 486.27 Mb containing 954 contigs with contig N50 = 2.36 Mb and 170 scaffolds with N50 = 59.10 Mb (Supplementary Table 3 and Supplementary Figure 3). Seven pseudochromosomes were anchored, covering 99.31% of the final assembly (Figure 1A and Supplementary Tables 3, 4). To further assess the quality of the genome assembly, Illumina paired-end reads were mapped to the final assembly. The mapping rate of 99.82% suggested the high uniformity of the sequencing. The Benchmarking Universal Single-Copy Orthologs (BUSCO) analysis showed that 94.58% of 1,440 plant lineage single-copy orthologs were present in the *T. arvense* assembly indicating the high completeness of the gene regions of the final assembly (Supplementary Table 5).

Based on genome assembly, we performed repetitive sequences annotation using a combination of *ab initio* and homology-based approaches. Overall, 65.86% of the genome assembly was identified as repeat regions, mainly including 57.92% retrotransposons, 2.20% DNA transposons, and 4.69% unclassified interspersed repeats (Table 1 and Supplementary Figure 4). Gene prediction was performed by a comprehensive strategy combining evidence-based and *ab initio* gene prediction after repeats masking of the genome. A total of 36,556 protein-coding genes were predicted, 99.61% of which were anchored to seven chromosomes in our genome assembly (Supplementary Table 4). The average length of the predicted genes was 1,861.9 bp, and each gene averagely harbored 4.46 exons with an average exon length of 232.8 bp (Supplementary Tables 4, 6). The functional annotation of protein-coding genes was achieved by searching against the Swissprot, RefSeq, InterPro, Pfam, and GO protein databases. Overall, 31,079 (85.02%) predicted genes were functionally annotated (Supplementary Table 7). We analyzed the global distributions of the genes and TEs on pseudochromosomes and found that the density of *Gypsy* LTR-retrotransposons was negatively correlated with gene density (Pearson correlation = −0.25, *P* = 1.128e-15). The densities of *Copia* LTR-retrotransposon, non-LTR retrotransposons (LINE and SINE), and DNA transposons were relatively low and their distributions were random (Figure 1A).

**TABLE 1:** Statistics of repeats contents in the *T. arvense* genome.

Type			Length (Mb)	Percent (% genome)	Percent (% all repeats)
All repeats			320.26	65.86	100.00
	Satellites		0.02	0.003	0.00
	Simple repeats		3.46	0.71	1.08
	Low complexity		0.83	0.17	0.26
	Small RNA		1.11	0.23	0.35
	Transposable elements		315.15	64.81	98.41
		Class I: retrotransposon	281.64	57.92	87.94
		LTR retrotransposon	276.88	56.94	86.46
		*Gypsy*	250.65	51.55	78.27
		*Copia*	14.59	3.00	4.56
		Unknown	11.64	2.39	3.63
		Non-LTR retrotransposon	4.76	0.98	1.49
		*SINE*	0.33	0.07	0.11
		*LINE*	4.44	0.91	1.38
		Class II: DNA transposon	10.69	2.20	3.34
		Unclassified interspersed repeats	22.82	4.69	7.12

### Gene and Genome Duplication

We identified paralogous genes in the *T. arvense* genome and then calculated the rate of synonymous substitution (*Ks*), which is defined as the number of synonymous substitutions per the number of synonymous sites, to estimate the age of duplication events. Based on the characteristics of paralogous copies, all paralogs were classified into four types of gene duplication by using MCScanX (Wang et al., 2012). Overall, dispersed duplicated genes accounted for the most with 33.12% of all duplicates followed by whole-genome duplication (WGD)/segmental, proximal, and tandem duplicates (TDs) accounting for 12.19, 10.33, and 9.51%, respectively (Supplementary Figure 5 and Supplementary Table 8). However, following the increment of time, the number and percentage of dispersed and WGD genes showed a downward trend. In contrast, the number and percentage of proximal and tandem duplicated genes increased continuously over time (Supplementary Figure 6 and Supplementary Table 9).

To investigate the genome evolution of *T. arvense*, we identified putative WGD events by analyzing the *Ks* distribution of synteny paralogs. We found that α WGD (*Ks* = 0.75) event was the most recent WGD event for *T. arvense*, and this WGD event was shared by *T. arvense* and three other members of Brassicaceae (*Arabidopsis thaliana, Brassica rapa*, and *Eutrema salsugineum*), except for *B. rapa* which experienced an addition of a whole-genome triplication (WGT) (*Ks* = 0.3) (Figure 1B; Wang et al., 2011). Conclusively, *T. arvense* has not undergone an additional species-specific WGD event after it diverged from *E. salsugineum* (Figure 1C).

### Phylogeny and Orthogroups Analysis

We first constructed the phylogenetic tree using 532 single-copy gene families from *T. arvense* and other eight angiosperms, including *Amborella trichopoda, Oryza sativa, Vitis vinifera, A. thaliana, A*rabidopsis *lyrata, B. rapa, Brassica oleracea*, and *E. salsugineum.* As shown in Figure 1C, *T. arvense* is most closely related to *E. salsugineum*. This result agrees with previous species relationships (Huang et al., 2016). Using this phylogenetic tree and three fossil calibrations, we estimated that *T. arvense* and *E. salsugineum* diverged from each other 9.7–15.4 MYA and that both species shared a common ancestor with *A*. *thaliana* and *A*. *lyrata* 15.1–24.0 MYA (Figure 1C).

We then identified orthogroups using OrthoFinder (Emms and Kelly, 2019) based on sequence similarity. The orthogroup was defined as the set of genes descended from a single gene in the last common ancestor from one or more species being considered (Emms and Kelly, 2015). We compared orthogroups among *T. arvense* and other three close relatives, including *E. salsugineum, A. thaliana*, and *A. lyrata*. A total of 18,250 *T. arvense* orthogroups were clustered, of which 15,249 orthogroups were shared with three other species and 944 were *T. arvense* specific. Unexpectedly, the *T. arvense* genome contains the most species-specific orthogroups among these four species (Figure 1D). Among these orthogroups, we further extracted significantly expanded orthogroups (referred to as “SEOs” thereafter), which were identified by computational analysis of gene family evolution (CAFE) based on the *P*-value associated with the gene family sizes between the extant species and the estimated ancestral nodes (De Bie et al., 2006). More surprisingly, the *T. arvense* genome possesses the most SEOs with a total of 345 orthogroups identified (*P* < 0.05) (Supplementary Table 10). Based on the GO annotations, the genes in these *T. arvense* SEOs are significantly enriched in “cysteine-type peptidase activity” (*P* = 1.7E-103) (Supplementary Table 11). Moreover, the GO summary indicated that the genes of SEOs in the *T. arvense* genome are related to growth and development and stress responses, e.g., “hormone biosynthetic process,” “signal transduction in response to DNA damage,” “positive regulation of multicellular organism growth,” and “ubiquitin-dependent protein catabolic process” (Figure 1E). Based on Kyoto Encyclopedia of Genes and Genomes (KEGG) annotations, these SEOs are highly enriched in DNA replication proteins, mismatch repair, DNA replication, homologous recombination, nucleotide excision repair, DNA repair and recombination proteins, aminobenzoate degradation, and circadian rhythm pathway (Supplementary Figure 7 and Supplementary Table 12).

### Long Terminal Repeat Retrotransposon Superfamily

We searched all kinds of repetitive sequences of the *T. arvense* genome. LTR-RTs are the most abundant type of TE, covering 56.94% of the genome, accounting for 86.46% of the total repeats component (Supplementary Figure 4). Among the LTR-RTs, the *Gypsy* superfamily was predominant, making up 51.55% of the genome, followed by the *Copia* superfamily accounting for 3.00% of the genome (Table 1). In order to test whether the richness of LTR/*Gypsy* is specific for the *T. arvense* genome, we applied the same criteria and methodology to search repeat sequences in the other four Brassicaceae species, *E. salsugineum*, *Schrenkiella parvula*, *B. rapa*, and *A. thaliana*. Expectantly, retrotransposons, especially the LTR/*Gypsy* superfamily, were the most abundant TEs in five Brassicaceae species genomes (Supplementary Figure 8). Among these five species, the *T. arvense* genome indeed had the highest percentage of LTR-RTs (56.94%) (Supplementary Figure 8), followed by 31.91, 20.48, 8.47, and 7.56% for the genome of *E. salsugineum, B. rapa, S. parvula*, and *A. thaliana*, respectively (Supplementary Table 13).

### Long Terminal Repeat Retrotransposon Family Identification, Amplification, and Divergence

**FIGURE 2:**
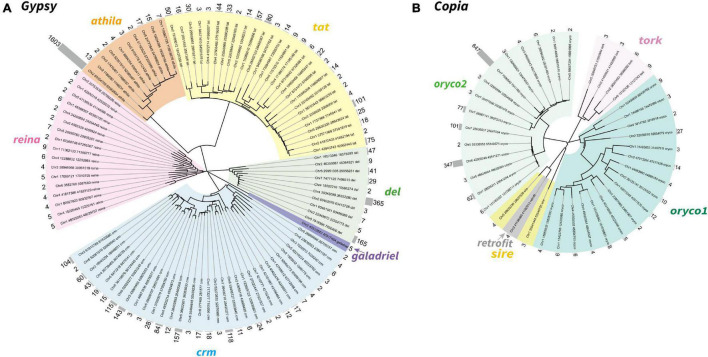
Neighbor-joining phylogeny of the LTR-RTs *Gypsy* **(A)** and *Copia* **(B)** superfamily in *T. arvense*. Different lineages were shown in clades marked in different colors. Each branch represents an LTR-RT family. The gray histogram indicates the copy number of each family (counted based on reverse transcriptase domains).

To further explore the proliferation of *Gypsy* and *Copia* superfamilies, we defined “family” in each superfamily based on the peptide sequence similarity of known reverse transcriptase (RT) domain from GyDB (Llorens et al., 2011) and following previous methods (Baucom et al., 2009). We then constructed the phylogeny of LTR-RTs and counted the copy number of families to investigate the activation of each family. Phylogenetic trees revealed that the *Gypsy* superfamily was grouped into six lineages, i.e., *del*, *galadriel*, *crm*, *reina*, *athila*, and *tat* (Figure 2A). The *Copia* superfamily in the *T. arvense* genome was basically grouped into five lineages, i.e., *oryco1*, *oryco2*, *tork*, *sire*, and *retrofit*, except that *sire* was nested within *retrofit* (Figure 2B). We identified a total of 4,110 copies for 98 families in six clades of the *Gypsy* superfamily. Specifically, the *crm* clade had the most families with 33, followed by the *tat* clade with 27 families. The copy number (1,630) of one family in the *athila* clade was the highest among all families in LTR-RT superfamilies. The *Galadriel* clade contains only one family with five copies. The *reina* and *galadriel* clades contain the least copy number of families (<10) (Figure 2A and Table 2). For the *Copia* superfamily, we identified a total of 1,422 copies for 38 families in five clades. However, most clades in the *Copia* superfamily possess few copies of each family, except that the *oryco2* clade has the average copy number of families larger than 10 (84.53 copies per family) (Figure 2B and Table 2).

**TABLE 2:** Numbers of families of *Gypsy* and *Copia* superfamily of LTR-RTs in *T. arvense* genome.

Superfamily	Clade ID	Number of families	Number of RT domains	Average number of copies per family
*Gypsy*	*athila*	10	1668	166.80
	*crm*	33	1049	31.79
	*del*	10	673	67.30
	*galadriel*	1	5	5.00
	*reina*	17	75	4.41
	*tat*	27	640	23.70
	TOTAL	98	4110	41.94
*Copia*	*oryco1*	16	122	7.63
	*oryco2*	15	1268	84.53
	*retrofit*	1	4	4.00
	*sire*	2	7	3.50
	*tork*	4	21	5.25
	TOTAL	38	1422	37.42

**FIGURE 3:**
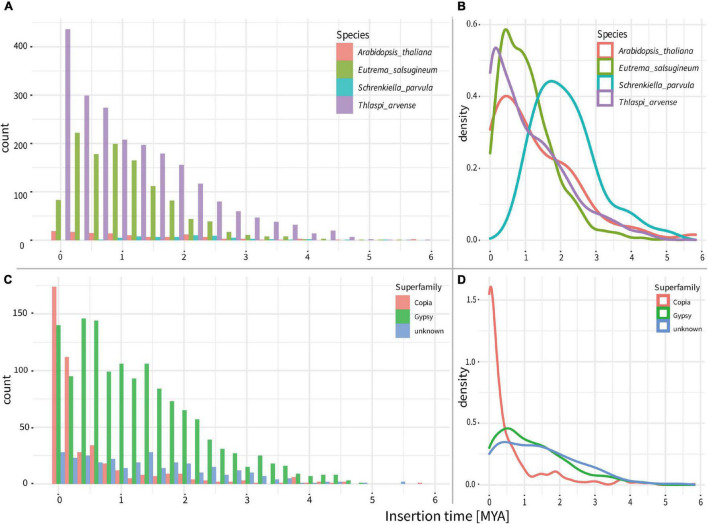
Ages of intact LTR-RTs in the genomes of *T. arvense* and other three close relatives of Brassicaceae. **(A)** Counts of all LTR-RTs ages in the *T. arvense* and other three close relatives. **(B)** Density lines of all LTR-RTs ages in the *T. arvense* and other three close relatives. **(C)** Counts of ages of different LTR-RT superfamilies in the *T. arvense*. **(D)** Density lines of ages of different LTR-RT superfamily in the *T. arvense*.

To investigate the evolutionary dynamics of LTR-RTs, we estimated the insertion time of all intact LTR-RTs. In comparison to the insertion times of LTR-RTs within three closely related species, *A. thaliana, E. salsugineum*, and *S. parvula*, the *T. arvense* genome possessed the most recently originated LTR-RTs, with the peak around 0.2 MYA (Figures 3A,B). Specifically, the origination of *Gypsy* in the *T. arvense* genome preferably occurs around 0.5 MYA with a successive proliferation since 4 MYA. However, the amplification of *Copia* increased abruptly since 1 MYA and reached the greatest density around 0.1 MYA (Figures 3C,D).

### Long Terminal Repeat Retrotransposons and Gene Duplication

Considering that 56.94% of the *T. arvense* genome was composed of LTR-RTs (Supplementary Table 13), we explored the impact of LTR-RTs on gene duplication. We identified 351 retrogenes and their corresponding parental genes (Supplementary Table 14) by integrating and improving previous strategies (Zhang et al., 2005; Zhu et al., 2009), which considered the retrogenes produced through alternative splicing mechanisms based on a recent study (Zhang et al., 2014; Supplementary Figure 9). Surprisingly, 78.92% of the retrogenes contain an intron(s). Comparing these intron-containing retrogenes to their parental genes, we identified that the intron-containing retrogenes were either emerged through alternative splicing mechanisms, e.g., intron retention or exon skipping (Supplementary Figures 10b,c, 14b), as described in previous studies in plants and animals (Zhang et al., 2014; Kim et al., 2017), or acquired intron(s) resulting in the variable structure of exons (Supplementary Figures 10b,f,g). Mapping all retrogenes and their parental genes on the *T. arvense* genome revealed a higher distribution of parental genes and their paralogs in Chr1 than in other chromosomes (Figure 1A track h). Additionally, integrating and improving previous strategies (Kim et al., 2017), which considered that the genes were retroduplicated if genes were fully contained within LTR-RTs, we identified 303 genes flanked by LTRs (Supplementary Table 15), including eight retrogenes and 295 genes located inside LTR-RTs, respectively.

Based on the association between annotated genes and the presence of LTRs, we categorized *T. arvense* annotated genes into four types. Type “A1,” “A2,” “A3,” and “B1” represent the retrogene flanked by LTRs (referred to as “LTR-retrogene” hereinafter), retrogene not flanked by LTRs (referred as “free retrogene”), non-retrogenes flanked by LTRs (referred as “LTR-gene”), and non-retrogene not flanked by LTRs (referred as “free gene”), respectively. We considered type A1, A2, and A3 genes all as retroduplicated genes, as they all were mediated by retrotransposons. Type A1 LTR-retrogenes were inferred to be produced by retrotransposition mediated by LTR-retrotransposons; type A2 free-retrogenes might be derived from retrotransposition mediated by non-LTR retrotransposons, or LTR-retrotransposons with sequences degradation; and type A3 LTR-genes, as identified in a recent study in hot peppers (Kim et al., 2017), were classified as retroduplication events mediated by LTR-retrotransposons. After removing 4,243 TE-like genes, the number of genes in each type are 8 (type A1), 343 (type A2), 295 (type A3), and 31,667 (type B1) among 32,313 annotated genes. By matching these four type genes to plant orthologs, the numbers of corresponding orthogroups are 8, 265, 211, and 20,576, respectively (Supplementary Table 16). Strikingly, orthogroups containing type A1 LTR-retrogenes show the highest proportion (50.00%) of SEOs followed by that of type A3 LTR-genes (27.96%) and A2 free-retrogene (7.20%). Orthogroups that contained non-retrotransposed genes (type B1) have a much low proportion (1.28%) of SEOs (Supplementary Table 16). These results imply that retroduplication contributed to the expansion of orthogroups.

**FIGURE 4:**
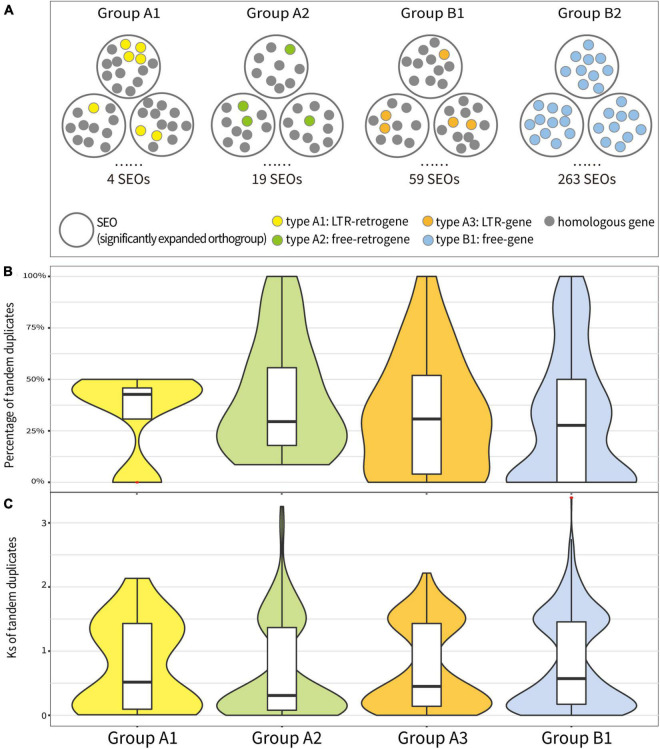
Analysis of tandem duplicated genes in significantly expanded orthogroups (SEOs) in Groups A1, A2, A3, and B1. **(A)** Conceptual diagram of analysis method for the tandem duplicated genes in SEOs in four groups. **(B)** The percentage of tandem duplicated genes of each SEO in four groups. **(C)** The Ks values of tandem duplicated genes in each SEO in four groups. Red asterisks represent outliers.

To understand how the retrotransposons affected the expansion of orthogroups, we analyzed SEOs that contained the aforementioned four types of genes and correspondingly categorized them as “Group A1,” “Group A2,” “Group A3,” and “Group B1.” In each SEO, we observed different percentages of TDs among these four groups. In *T. arvense* genome, SEOs that contained LTR-retrogenes (Group A1) had the highest percentage of TDs (42.71%) followed by Group A3 (30.77%), Group A2 (29.51%), and Group B1 (27.66%) based on the sample median (Figure 4B). The mean percentage of tandem duplicated genes also showed that SEOs that contained genes produced by retroduplication (Group A1, A2, and A3) had more TDs than SEOs that contained only non-retrotransposed genes (Group B1) (Supplementary Tables 17, 18). Besides, the *Ks* values of the TDs in Group A2 were medially smallest as 0.31, following Group A3 (0.45), Group A1 (0.52), and Group B1 (0.57) (Figure 4C and Supplementary Tables 17, 19). The comparison analysis among Groups A1, A2, A3, and B1 indicated that SEOs that contained retroduplicated genes (retrogenes or genes inside LTR-RTs) possessed more and younger tandem duplicated genes than SEOs that contained only non-retrotransposed genes (Figure 4).

Furthermore, we selected Group A1, A2, and A3 SEOs that possessed more than 40% of TDs and checked whether the three types of genes in SEOs are TDs themselves. We found that 66.67% of LTR-retrogenes in Group A1, 70.37% of free-retrogenes in Group A2, and 85.11% of LTR-genes in Group A3 were TDs themselves (Supplementary Table 20). We calculated and compared the *Ks* values of the LTR-retrogenes/free-retrogenes with their respective parental genes and with their respective TDs. Seventy-five percent of the LTR-retrogenes/free-retrogenes being TDs themselves underwent the tandem duplication after the retroduplication (Supplementary Figure 11). Additionally, a comparison of *Ks* values for all TDs and retrogenes with their parental genes in Group A1 and A2 showed that the divergence time of TDs was averagely younger than the ones of retrogenes (Supplementary Figure 12). The above results suggest that RNA-based duplication (retroduplication) triggered the subsequent recent tandem duplication of homologous genes.

### Analysis of Expanded Gene Families Induced by Retroduplication

For SEOs that contained genes mediated by retroduplication (Group A1, A2, and A3) and possessed more than 40% of TDs, we characterized the domains of these genes and then analyzed the related gene families consisting of orthogroups harboring the same protein domains. All these families were expanded in the *T. arvense* genome by retroduplication followed by tandem duplication of homologous genes. Nine families were involved in plant growth and development and stress resistance, and four families were related to TE, e.g., transposase family (Supplementary Table 21). We carried out a phylogeny analysis of the nine gene families with orthogroups among *T. arvense*, *E. salsugineum, A. thaliana*, and *A. lyrata*.

**FIGURE 5:**
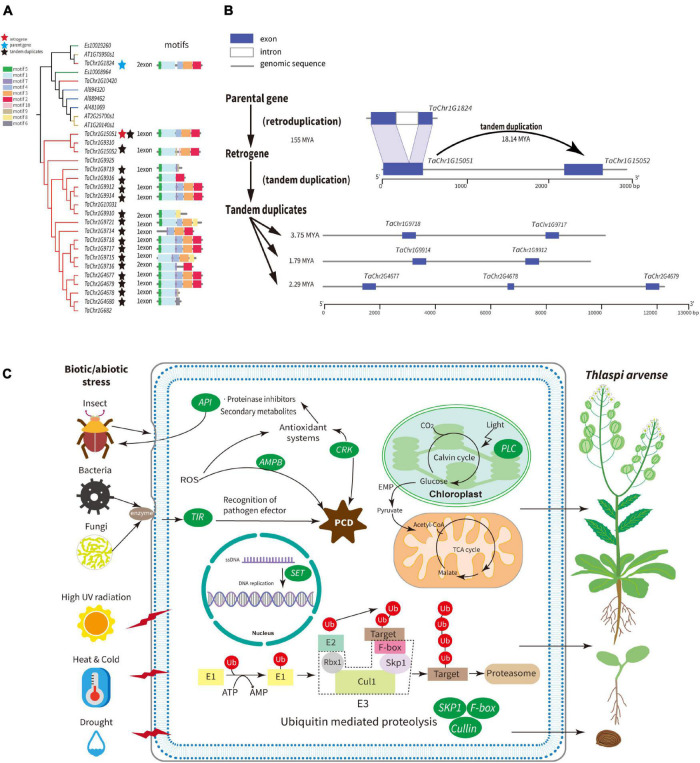
Schematic summary of adaptive strategies of *T. arvense.* **(A)** Phylogenetic relationships, motifs of the *SKP1* gene family between *T. arvense* (gene id with “TaChr”), *E. salsugineum* (gene id with “Es”), *A. lyrata* (gene id with “Alyrata”), and *A. thaliana* (gene id with “AT”). The blue star indicates the parental gene; red star indicates the retrogene; dark stars indicate tandem duplicates. **(B)** Models for the evolution of *SKP1* genes, as one example family that induced by the mechanism that retroduplication followed by subsequent tandem duplication of homologous genes. **(C)** Expanded gene families induced by RNA-based and tandem gene duplication are shown in green ovals, which are related to growth and development and stresses responses in *T. arvense* genome.

The *SKP1* gene family contains the SEO belonging to Group A2, whose SEO contained free-retrogenes. Two clades are clearly clustered and defined as A and B (Supplementary Figure 13). The genes in clade B are mostly specific to *T. arvense* and contain the majority of *SKP1* genes. Therefore, we specifically examined a few *SKP1* genes in clade B. For example, *TaChr1G15051* is a retrogene that lost one intron compared with its parental gene *TaChr1G1824*, and both share the same motifs (motif 1–5 in Figure 5A). Moreover, *TaChr1G15051* and *TaChr1G15052* are paralogous derived from tandem duplication. The *Ks* analysis showed that retrogene *TaChr1G15051* diverged from its parental genes around 155 MYA and TD gene about 18.14 MYA (Figure 5A and Supplementary Tables 14, 18). Besides, we found that 80.95% of the *SKP1* genes in the *T. arvense* species-specific clade were tandem duplicated genes and were diverged from each other about 7.41 MYA with the most recent divergence time around 1.79 MYA (Supplementary Table 19). Collectively, the *SKP1* family is an ideal model for understanding the family expansion mechanism mediated by retroduplication followed by tandem duplication.

The *SET* family contains the Group A1 SEO. The parental gene *TaChr3G257* is flanked by LTRs and is the TD with retrogene *TaChr3G258*. *TaChr3G257* gene also gave rise of an additional free-retrogene *TaChr3G225* and an LTR-retrogene *TaChr3G234*. By comparing their gene structures, we show that these three retrogenes emerged through exon skipping alternative splicing mechanisms, sharing the same motifs, and diverging from the parental gene 9.36, 8.95, and 3.54 MYA, respectively (Supplementary Figure 14). Other TDs in this family only possess parts of the motifs of the parental gene.

Among other seven gene families that involved plant growth, development, and stress responses, the *AMP-binding* (*AMPB*) family and *Cu_bind_like* (*PCL*) family which contain a plastocyanin-like domain, both possess SEOs contained free-retrogenes and are expanded by TDs (Supplementary Figures 15, 16). The *F-box* and *leucine-rich repeat* family contained SEOs belonging to Group A2 and A3. The retrogene *TaChr3G404* was separated from the parental gene *TaChr1G14282* around 56.04 MYA, and this parental gene was tandemly duplicated after its retroduplication (Supplementary Figure 17 and Supplementary Table 19). The LTR-gene (*TaChr6G576*) in the *F-box* family was tandemly duplicated very recently (Supplementary Table 19). The last four families, i.e., *Cullin, Cysteine-rich receptor-like kinases* (*CRK*), *Aspartic proteinase inhibitors* (*API*), and *Toll/interleukin-1* receptor (*TIR*), all contain LTR-genes in SEOs belonging to Group A3 and are also expanded by TDs (Supplementary Figures 18–21). Lastly, for each of these nine gene families, we used previously described transcriptomes of *T. arvense* to obtain expression levels (transcripts per million, TPM) for all retroduplicated genes and tandem duplicated genes that were younger than retroduplicated genes or had divergence time less than 6 MYA (Dorn et al., 2013; Thomas et al., 2017). Overall, 78% of these genes showed expression, especially one tandem duplicated gene (*TaChr1G15052*) in the *SKP1* family, and one free-retrogene (*TaChr4G2027*) in the *AMPB* family showed much higher expression levels in nectary tissues (Supplementary Table 22).

## Discussion

We presented a high-quality assembly of the *T. arvense* genome by combining PacBio SMRT, Bionano optical mapping, Hi-C, and NGS sequencing technologies. The quality of the genome assembly is substantially improved compared with the one mainly based on NGS sequencing, e.g., the scaffold N50 length of our assembly had been improved more than 420-fold of that based on NGS sequencing (Supplementary Table 3; Dorn et al., 2015). Compared with another recently reported genome sequence of *T. arvense* based on Nanopore, NGS, and Hi-C sequencing technologies (Geng et al., 2021), we observed similar overall quality. For example, there is a < twofold difference in the N50 length of contigs in two assemblies (Supplementary Table 3). The total assembly size in the previously published assembly is larger and closer to the estimated genome size (539 Mb) from flow cytometry (Johnston et al., 2005; Supplementary Table 3), which could be due to genome size variation within species stemming from the accumulation of repetitive elements (Biemont, 2008; Diez et al., 2013; Zhang J. et al., 2020), the difference in sequencing depth (Supplementary Table 3), and/or the longer sequence reads from Nanopore sequencing technology but higher error rate (Lu et al., 2016). However, our final genome assembly has a higher anchoring rate on seven pseudochromosomes (99.31 vs. 90.08%) (Supplementary Table 3). Our Hi-C contact map suggested a cleaner background of the map, where all bins were clearly divided into the seven pseudochromosomes without signal-noise interaction detected between different chromosomes (Supplementary Figure 22).

Genome comparison showed that the genome size of *T. arvense* (est. 486 Mb) was at least twice larger than that of its close relatives *E. salsugineum* (243 Mb), *Noccaea caerulescens* (267 Mb), and *Thellungiella parvula* (137 Mb), where they all share the same number of chromosomes (2*n* = 14) (Supplementary Figure 3; Dassanayake et al., 2011; Yang et al., 2013; Mandakova et al., 2015). To elucidate the causes and consequences of genome size variation in *T. arvense* and its closely related species, it is crucial to have detailed information about its genomic architecture. Therefore, we set out to investigate if the genome size difference could be caused by the expansion of specific genetic elements. Genome-wide paralogous comparative analysis showed that *T. arvense* did not experience a recent species-specific WGD event. However, *T. arvense* possessed the most SEOs when compared with *E. salsugineum*, *A. thaliana*, and *A. lyrata*. Thus, this may partially contribute to *T. arvense* genome size expansion. Retrotransposons are the main components of plant genomes and their activations frequently result in their duplication and insertion, leading to an increase in genome size (Bennetzen et al., 2005; Piegu et al., 2006). Based on the high-quality chromosome-scale genome assembly with precise genome structures, our in-depth repeats analysis revealed that LTR-RTs accounted for most of the *T. arvense* genome. LTR-RT family identification showed that all lineages of *Gypsy* and *Copia* superfamilies in *T. arvense* were also found in the *Aegilops tauschii* genome (Zhao et al., 2017). Specifically, the *Gypsy* superfamily accounts for over half the size of the *T. arvense* genome assembly, and the *athila* and *crm* lineages made a great proportion to the proliferation of the *Gypsy* superfamily (Figure 2A and Table 2). For further study of the evolution of LTR-RTs, nested LTR-RTs and relevant pipelines should be considered (Jedlicka et al., 2019; Lexa et al., 2020). Overall, LTR-RT proliferation largely contributes to the enlargement of the *T. arvense* genome size, which is consistent with a recent report (Geng et al., 2021).

Genome restructuration mediated by TE activity is essential for the stress response of hosts, which can facilitate the adaptation of species to changing environments (McClintock, 1984; Bourque et al., 2018; Huang et al., 2020). Our analysis found that LTR-RTs had been recently more active in *T. arvense* than those in the other three close relatives of Brassicaceae since *T. arvense* diverged from *E. salsugineum*. Especially, the *Gypsy* superfamily has been accumulated steadily since 4 MYA, and the number of *Copia* increased sharply since 1 MYA. The insertion events of *Gypsy* and *Copia* LTR-RT reached the peak around 0.5 and 0.1 MYA in *T. arvense*, respectively (Figures 3C,D). Intriguingly, the time of the fastest LTR-RTs accumulation is consistent with the time that QTP unique haplotypes of *T. arvense* were separated from others during the middle Pleistocene (An et al., 2015). *T. arvense* plants survived from the glacial–interglacial cycle through molecular or phenotypic plasticity during the Quaternary, especially in QTP regions, just like most plants have been experienced (Nicotra et al., 2010). Consequently, the expansion of LTR-RTs provided great genetic diversity for *T. arvense* phenotypic plasticity to confront extreme environmental conditions.

To get a better understanding of how retrotransposons affect genome evolution, we identified 351 retrogenes and 303 genes flanked by LTRs in *T. arvense* genome. Compared with the 251 retrogenes and three retrogenes flanked by LTRs identified in the *A. thaliana* genome (Abdelsamad and Pecinka, 2014; Zhu et al., 2016), more retrogenes were identified in the *T. arvense* genome, which might result from the abundance of retrotransposons in the *T. arvense* genome. More significantly, our data show that retroduplication, especially retroduplication mediated by LTR-RTs, contributed to the expansion of orthogroups (Supplementary Table 16). To further unravel the mechanism of the retrotransposons impacting the gene duplication, we examined the activities and number of orthogroups associated with genes mediated by retroduplication. Firstly, our data demonstrated that the number and percentage of tandem duplicated genes increased continuously over time in the *T. arvense* genome (Supplementary Figure 6). Secondly, if orthogroups contained any kind of retroduplicated genes, these orthogroups possessed more and younger tandem duplicated genes than those that only contained non-retrotransposed genes (Figure 4). A similar phenomenon was reported in Solanaceae family plants where some disease resistance retroduplicated genes gained new function *via* subsequent tandem duplication (Kim et al., 2017), but at a family level rather than a species level. Our whole-genome analysis collectively showed that retroduplication facilitates the subsequent tandem duplication of homologous genes.

The alteration of gene family size facilitates the successful colonization of extreme environments by various eukaryotes (Ma et al., 2013; Huang et al., 2020; Zhang Z. et al., 2020). Populations of *T. arvense* surviving in the QTP region were exposed to dynamic environments driven by mountain building, Quaternary glacial cycles, and the intensification of the Asian monsoon (Ding et al., 2020). Furthermore, an extreme environment resulting in abiotic stresses and biotic stresses could affect the growth of *T. arvense.* We analyzed the expanded gene families induced by the mechanism of the retroduplication followed by tandem duplication of homologous genes (Supplementary Table 21). All these families contain genes that are related to retroduplication and tandem duplication. We then specifically examined the molecular and cellular functions of these expanded gene families as well as their association with the development and stress responses of *T. arvense* plants.

Nine gene families including *SKP1*, *Cullin*, *F-box*, *SET*, *AMPB*, *PCL*, *API*, *CRK*, and *TIR* were expanded in the *T. arvense* genome by the mechanism of the retroduplication followed by the tandem duplication of homologous genes (Supplementary Figures 13–21), which might synthetically contribute to its survival in the harsh environment in the QTP regions. Ubiquitin (Ub)-mediated regulation is one of the fundamental mechanisms for degradation and protein signaling in eukaryotes (Hua and Vierstra, 2011), which plays crucial roles in vegetative/flower development and stress signaling. The ubiquitin molecule needs enzymes to attach to the target protein, and the *SCF* (*SKP1/Cullin/F-box*) protein complexes formed by *SKP1*, *Cullin*, *Rbx1*, and *F-box* proteins are one type of E3 ubiquitin ligases (Figure 5C). In our study, *SKP1*, *Cullin*, and *F-box* families in the *T. arvense* genome were all expanded by the mechanism of the retroduplication followed by the tandem duplication of homologous genes (Figure 5 and Supplementary Figures 17, 18). The *SET* domain proteins are involved in DNA replication and globally influence plant development (Thorstensen et al., 2011); *AMPB* domain proteins widely exist in various plant species, and some members of this family were related to programmed cell-death induced by reactive oxygen species (ROS) (Liu H. et al., 2016); Phytocyanins are ancient blue copper-binding proteins in plants that function as electron transporters and possess the plastocyanin-like (*PCL*) domain. The *PCL* gene family also plays an important role in plant development and stress resistance (Xu et al., 2017); Plants *API* genes have functions in protecting plant proteins against exogenous proteases synthesized by parasitic viruses, bacteria, and insects and are highly transcribed and translated in seeds and fruits (Volpicella et al., 2011); The *CRK* gene family, which harbors salt stress response/antifungal domain (Supplementary Table 21), plays crucial roles in plant responses to biotic and abiotic stresses and most of them are regulated by ROS, common signaling molecules produced in response to various stresses in plants (Gu et al., 2020); the *TIR* domain is the signature signaling domain of Toll-like receptors and their adaptors. The *TIR* gene family mediate disease resistance in plants (Essuman et al., 2018). Two studies have shown that gene families rich in retroposed genes are subject to tandem duplication (Jiang et al., 2010; Liu Z. et al., 2016). In addition, this pattern of family expansion is also shown in four gene families related to transposase-associated function in the *T. arvense* genome, implying that retrotransposons might have impacts on other TE’s evolution.

Taken together, these specific gene family expansions in the *T. arvense* genome, which are associated with plant growth and development, abiotic, and biotic stress responses, appear to have been a driving force for *T. arvense*’s adaptability to extreme environments, contributing to its worldwide distribution (Figure 5C). Studies have shown that LTR-RTs can be activated under conditions of biotic and abiotic stress (Feschotte et al., 2002), and TDs have a more important role in stress adaptations than other types of gene duplications (Oh et al., 2012). The two flanking LTRs, the recognizable characteristics of LTR-RTs, not only provide regulatory motifs for paralogous genes functionalization but also serve the avenue for ectopic recombination and unequal crossing-over (Stritt et al., 2020) that facilitate the tandem gene duplication (Panchy et al., 2016).

The strong adaptability of *T. arvense* has been described based on the evidence from population genetics profiling (Geng et al., 2021) and phylogeographic data analysis (An et al., 2015). Here, based on the comparative genomic analysis, we provided the molecular mechanism of how the *T. arvense* adapted to the harsh environment. Collectively, our data and results suggested that retroduplication and the subsequent tandem duplication of plant growth/development/stress responses related genes might be one of the key strategies for the rapid adaptive evolution of *T. arvense*. As the phenomenon that some gene families that contained retropositioned genes are subject to having TDs was also found in soybean and within Solanaceae and Brassicales, we hypothesize that retrotransposons mediated mechanism was one strategy for plants adaptive evolution. Overall, the high-quality assembly of the *T. arvense* genome provides insights into the mechanisms of plant adaptation to extreme environments and provides fundamental resources for comparative genomics studies and genetic improvement.

## Materials and Methods

### Plant Materials and Genome Size Estimation

Seeds [voucher number, LiJ461 (KUN)] of *T. arvense* were collected from the Tibetan Autonomous Prefecture of Garzê, Sichuan, China, whose habitat is in a high mountain meadow. These seeds of *T. arvense* were obtained from the Germplasm Bank of Wild Species in the Kunming Institute of Botany. They were planted and cultivated in the greenhouse at the Kunming Institute of Botany, Chinese Academy of Sciences. The tender leaves of plants were used for genome sequencing. We generated about 234 million 100-bp paired-end Illumina reads (−46× coverage), sequenced on the Illumina HiSeq 2000 (Illumina, 5200 Illumina Way, San Diego, CA, United States) platform. The base quality was assessed with FastQC^1^ before and after data cleaning. We estimated the genome size and heterozygosity rate by a *k*-mer distribution analysis with PBjelly (English et al., 2012) and GenomeScope (*k* = 41) (Vurture et al., 2017), using sequenced Illumina reads.

### Single-Molecule Real-Time PacBio Genome Sequencing and *de novo* Assembly

The tender leaves of one single plant were acquired for PacBio SMRT sequencing. SMRT sequencing libraries were constructed using the PacBio protocol “Procedure & Checklist – 20 Kb Template Preparation Using BluePippin™ Size-Selection System.” The genome was sequenced employing four SMRT cells on the PacBio Sequel platform (Pacific Biosciences, CA, United States) by sequencing provider Wuhan Nexomics.^2^ We obtained 28.11 Gb of the raw sequence of the targeted genome 50-fold coverage. Furthermore, a total of 27.99 Gb subreads with a mean read length of 8,886 bp were generated.

We used the Canu (Koren et al., 2017) pipeline to assemble the reads into contigs with high-sensitivity parameters (corOutCoverage = 80). The contamination of contigs from bacterial, viral, human, and plasmid genomes was eliminated using the Basic Local Alignment Search Tool (BLAST) against the corresponding NR sub-database. A total of 13 contigs was removed as plasmid sequences and no other contamination was found.

### BioNano Optical Map Sequencing and Hybrid Scaffold Construction

High-molecular-weight DNA was isolated from young leaves tissue. DNA was labeled at Nt.BspQI sites (Label Density 13.66/100 Kb) using the SaphyrPrep kit. Labeled DNA samples were loaded and run on the Saphy system (BioNano Genomics, CA, United States) (service provided by Wuhan Nexomics, see text footnote 2). A 600-fold coverage (323 Gb) optical map of the genome was produced with single labeled molecules above 150 Kb in size.

Molecules collected from BioNano chips were *de novo* assembled into consensus physical maps by BioNano Solve 3.0^3^ using “optArguments_haplotype_saphyr.xml” and using the Canu assembled contigs to obtain the noise parameters. The hybrid scaffolds were created by aligning and merging these optical maps and previous curated sequence contigs by RefAligner of BioNano Solve 3.0 (-r RefAligner -o 150k -f -B 2 -N 2 -y).

### Hi-C Sequencing and Pseudomolecules Construction

After fixing cells with formaldehyde lysed and digesting the cross-linked DNA with *Dpn*II, the Hi-C libraries were constructed and sequenced on the HiSeq X Ten platform. Overall, 275 million 150-bp paired-end Illumina reads were produced. To assemble to a chromosome level, the Hi-C reads were aligned to the draft assembly by running the “bwa aln” algorithm, and the paired-end reads which were uniquely mapped onto the draft assembly scaffolds were finally grouped into seven chromosome clusters using the Lachesis software (RE_SITE_SEQ = GATC, CLUSTER_N = 7) (Burton et al., 2013).

### Contigs and Pseudomolecules Polishing

After the *de novo* assembly, we used blasr^4^ and Arrow^5^ to do polishing of the draft assembly with SMRT reads, and we also used Pilon^6^ with Illumina short reads to do the correction. The DNA used for Illumina sequencing was extracted from the same genotype of the leaf tissue that has been used for SMRT PacBio sequencing.

After constructing pseudomolecules, PBJelly^7^ (English et al., 2012) was used to fill gaps using SMRT raw reads with default parameters. Subsequently, the final assembly was polished again using Arrow. Lastly, we performed sequence error correction again with the Pilon pipeline after aligning reads to assembly with BWA^8^ mem algorithm and parsing with SAMtools (Li et al., 2009).

### Genome Assembly Quality Assessment

The final assembly had 1,282 gaps. The Illumina paired-end reads were mapped to the final assembly using BWA to evaluate the completeness of the assembly and the uniformity of the sequencing. The mapping rate was 99.26%, demonstrating that our assembly results contain almost all the information in the reads. Then we used BUSCO to evaluate the completeness of the gene regions.

### Repeat Annotation

For the *ab initio* predictions, we used three pipelines together to build a *de novo* repeat library, RepeatModeler for all kinds of repeats.^9^ LTRharvest (Ellinghaus et al., 2008) and LTR_retriver (Ou and Jiang, 2018) are used for the identification of LTR-retrotransposons. LTR_retriver got LTR-RT models from structural detection of LTRharvest. LTRharvest was set by requiring LTRs with 90% identity with the presence of the canonical/typical motif “TGCA.” For evidence-driven prediction, we used the RepeatDatabase module of RepeatMasker (see text footnote 9) to build a RepBase “seed plant” repeat library. We applied Repeatmodeler (see text footnote 9) to build *T. arvense* species-specific TEs library. Then, we combined these libraries and ran RepeatMasker on the assembly (default parameters) to identify and mask the repetitive sequences in the *T. arvense* genome. The same method of repeat annotations was applied to genomes of *A. thaliana* (version TAIR10), *E. salsugineum* (version 1.0, accession GCA_000478725.1 at NCBI GenBank), *S. parvula* (version 1.0, accession GCA_000218505.1 at NCBI GenBank), and *B. rapa* (version 3.0, accession number GWHAAES00000000 at Genome Warehouse database).

### Gene Prediction

After masking the repetitive sequences of the genome as described above, we identified protein-coding gene models using the FGENESH++ pipeline (Softberry Inc., Mount Kisco, NY, United States) with parameters trained with *A. thaliana* gene models. A transcriptome of *T. arvense* assembled from Illumina RNA-seq reads was used to facilitate the gene prediction with transcriptome evidence (Dorn et al., 2013). The *de novo* predicted gene model correction was performed by comparing all known plant protein sequences from the NCBI NR database.

### Gene Function Annotation

Using BLASTP (*E*-value 1e-5), the functional annotation of protein-coding genes was implemented according to the reciprocal best hit (RBH) of the alignments against two integrated protein sequence databases: SwissProt (UniProt database) and the NCBI non-redundant RefSeq protein database.^10^ The InterProScan software was used to annotate the protein domains by searching against the InterPro (Jones et al., 2014), to obtain the GO terms corresponding to the InterPro entries, and get pathways in which genes might be involved. Moreover, these were assigned by BLAST against the KEGG databases, with an *E*-value cut-off of 1e-5. Besides, the GO terms were obtained by using eggnog (Huerta-Cepas et al., 2019) and the KEGG annotations were obtained from the online KEGG automatic annotation server (KAAS)^11^ (Moriya et al., 2007).

### Orthogroup and Phylogeny Analysis

OrthoFinder (version 2.4.1) (Emms and Kelly, 2019) was used to identify orthogroups based on sequences similarity. Removing 4,243 genes highly similar to TE-like transposase which only had RT domains by gene function annotation, we used OrthoFinder (Emms and Kelly, 2019) to construct a set of 32,313 protein-coding genes into orthogroups. CAFE (version 4.2) was applied to identify the gene family’s expansion and contraction (De Bie et al., 2006). The GO and KEGG enrichment analysis of genes were conducted by clusterProfile (Yu et al., 2012). The GO enrichment of the expanded gene families of *T. arvense* was summarized using REVIGO (Supek et al., 2011), only showing GO categories that were significantly enriched (*P* < 0.05). Single-copy orthogroups were used to reconstruct the phylogeny. Multiple sequence alignment of protein sequences for each single-copy orthogroups was performed by multiple sequence comparisonbylog-expectation (MUSCLE) with default parameters (Edgar, 2004). The divergence time between species was estimated using MCMCTREE of PAML (Yang, 2007) (“correlated rates”; “JC69” model; burnin = 20,000,000, nsample = 200,000 and sampfreq = 1,000) with three calibration points, i.e., *Arabidopsis* origination time (4.8–9.8 MYA) (Guo et al., 2017), *B. rapa* and *B. oleracea* divergence time (2.0–3.2 MYA) (Kumar et al., 2017), and angiosperms origination time (167.0–199.0 MYA) (Stull et al., 2021).

### Paralog and Whole-Genome Duplication Analysis

We performed all-vs-all paralog analysis in *T. arvense* and other three relatives (*E. salsugineum*, *B. rapa*, and *A. thaliana*) genomes respectively by using BLASTP with RBHs. RBHs are defined as reciprocal best blast matches with an e-value threshold of 1e-7, length of RBHs longer than 100aa, and c-score (BLAST score/best BLAST score) larger than 0.3 (Guo et al., 2018). Based on the alignment produced by the MUSCLE program (Edgar, 2004), the synonymous substitution rate (*Ks*) for paralogous gene pairs was calculated using the paraAT 2.0 pipeline (Zhang et al., 2012). MCScanX (Wang et al., 2012) was used to classify types of duplicated genes and do synteny analysis within the genome with default parameters.

### Long Terminal Repeat Retrotransposon Family Analysis

We applied LTRharvest (Ellinghaus et al., 2008) and LTR_retriver (Ou and Jiang, 2018) to get non-redundant rexamplers of LTR-RTs in the *T. arvense* genome. All known RT domains were downloaded from GyDB^12^ (Llorens et al., 2011). We then blasted all known RT domains against the non-redundant LTR-RTs exemplars of the *T. arvense* genome (-outfmt 5, -max_target_seqs 1, -max_hsps 1, and length ≥ 200 bp) and used python scripts to extract the RT domains exemplars for each family and blasted them to the whole *T. arvense* genome. Sequences showing ≥ 80% similarity of the domain were grouped into one family as what was defined in the maize genome (Baucom et al., 2009). For each superfamily of LTR-RTs, the RT domain exemplars of families were aligned with MUSCLE for multiple sequence alignments (default parameter settings). Neighbor-Joining (NJ) trees were constructed with MEGA7 [Bootstrap (BP): 1,000 duplicates; pairwise deletion] (Kumar et al., 2016). For family naming, LTR-RT families are designated with the format “Chr_start_end_XXX” where XXX is the designation for the family, Chr is the source chromosome, and start and end are the coordinates. Genomic similar RT domains were non-redundant between families by using shell scripts. The Interactive Tree Of Life (iTOL),^13^ an online tool, was used for the display, manipulation, and annotation of phylogenetic trees (Letunic and Bork, 2019).

### Long Terminal Repeat Retrotransposon Age Estimation

Time of insertion of the LTR-RT was implemented by TLR-retriever (Ou and Jiang, 2018), which calculated the flanking LTR sequences of an intact LTR-RT by measuring the divergence between the LTRs. Based on the neutral theory, this divergence value (hereafter K) was used to calculate the LTR-RT age with the formula T = K/2μ, where substitution rates (μ) of 7 × 10^–9^ substitutions per site per year was used for *A. thaliana* (Zhang et al., 2019) and 9.1 × 10^–9^ for other three species (*E. salsugineum*, *S. parvula*, and *T. arvense*) which are closer to *B. rapa* (Park et al., 2019).

### Identification of Retrogenes and Genes Flanked by Long Terminal Repeats

To identify retrogenes in *T. arvense* genome, we refined the previous strategy (Zhang et al., 2005; Zhu et al., 2009). The flow chart of retrogenes identification is shown in Supplementary Figure 9. The scripts can be found at https://github.com/YantingHu/retrogenes_identification. After removing 4,243 TE-like genes which only had RT domains annotated by the InterProScan software (Jones et al., 2014), we did an all-to-all blastp of the remaining 32,313 genes using BLASTP. Gene pairs with similarity were retained for a further blastn, in which gene pairs with only one hit from DNA-based duplication were discarded. Then, we applied scripts to retain gene pairs in which at least two parental exons connect in one exon of the retrogene based on sites analysis (± 19 bp exon boundary shifts). Tandem duplication events of putative retrogenes with their parental genes were discarded. The numbers of exons were analyzed between the putative retrogene and the corresponding parental gene. Simultaneously we manually checked the structure of all retrogene candidates. For genes flanked by LTRs, we used LTR_retriever (Ou and Jiang, 2018) to search genes with flanking eight Kb sequences for LTRs. Genes located within a predicted LTR-RT by LTR_retriever were retained for further check. RepeatMasker was applied for false-positive checking and flanking eight Kb sequences of genes without annotations as LTR-RTs were discarded. In addition, because of the rapid deletion of LTR-RTs, we performed an additional identification of genes flanked by LTRs using the annotated repeats including the partial LTR-Rs generated by RepeatMasker, which was applied in a previous genome level study (Kim et al., 2017). As in Kim et al. (2017), we reasoned that if genes were fully contained within LTR-RTs annotated by RepeatMasker, the genes were retroduplicated.

### Analysis of Tandem Duplicated Genes in Gene Families

All orthogroups and SEOs were identified, which was described in the aforementioned “Orthogroups and phylogeny analysis” methods. Gene families were defined as the ones that consisted of orthogroups harboring the same protein domains. Tandem duplicated genes were defined if the genes were adjacent to each other in the same chromosome or near each other but separated by one gene in one orthogroup. Proximal duplicated genes were defined if the gene copies are closely located on the same chromosome and near each other separated by 2–19 genes in one orthogroup. WGD duplications were predicted by MCScanX (Wang et al., 2012). The synonymous substitution rate (*Ks*) for paralogous gene pairs was calculated using the paraAT 2.0 pipeline (Zhang et al., 2012). The MEME online software^14^ was used to analyze the motif of these families’ members and GSDS 2.0^15^ was used to display gene structures. The iTOL (see text footnote 13) was used for the display of phylogenetic trees (Letunic and Bork, 2019). RNA-seq reads were downloaded from the SRA database of NCBI with accessions PRJNA183634, PRJNA379465, and PRJNA388539. The reads were preprocessed to remove contaminating sequences and then aligned to the assembled genome using HiSat (Kim et al., 2015). Reference-guided assembly was performed and the non-redundant set of transcripts were merged using StringTie (version 1.3.3) (Pertea et al., 2015).

## Data Availability Statement

The datasets presented in this study can be found in online repositories. The names of the repository/repositories and accession number(s) can be found below: http://bioinfor.kib.ac.cn/THLASPI/, TAGP20172021; https://ngdc.cncb.ac.cn/, PRJCA006550.

## Author Contributions

CZ and YH designed the research. YH, LL, and DG planted the research materials and performed the *de novo* genome construction. YH and XW carried out the gene prediction and annotation. XW and JP constructed the species phylogeny tree. YH implemented the genome structure comparison, evolutionary analyses of TEs and genes, and retroduplication analyses, and prepared the figures and tables. RL performed transcriptome assembly and merged the non-redundant set of transcripts. YH, CZ, and GJ fulfilled the comparison between four groups. YH, CF, and CZ wrote the manuscript. All authors read and approved the final manuscript.

## Conflict of Interest

The authors declare that the research was conducted in the absence of any commercial or financial relationships that could be construed as a potential conflict of interest.

## Publisher’s Note

All claims expressed in this article are solely those of the authors and do not necessarily represent those of their affiliated organizations, or those of the publisher, the editors and the reviewers. Any product that may be evaluated in this article, or claim that may be made by its manufacturer, is not guaranteed or endorsed by the publisher.

## Footnotes

1
https://www.bioinformatics.babraham.ac.uk/projects/fastqc/

2
http://www.nextomics.cn

3
http://bnxinstall.com/solve/Solve3.2.1_04122018.tar.gz

4
https://github.com/PacificBiosciences/blasr

5
https://github.com/PacificBiosciences/GenomicConsensus

6
https://github.com/broadinstitute/pilon/releases/

7
http://www.winsite.com/Home-Education/Science/PBJelly/

8
https://github.com/lh3/bwa

9
http://www.repeatmasker.org

10
http://www.ncbi.nlm.nih.gov/refseq/

11
https://www.genome.jp/kaas-bin/kaas_org

12
https://gydb.org/index.php/Collection_MRC

13
https://itol.embl.de

14
http://meme.nbcr.net/meme/cgi-bin/meme.cgi

15
http://gsds.gao-lab.org/

## Abbreviations

LTRs: long terminal repeats
LTR-RTs: long terminal repeat retrotransposons
TEs: transposable elements
QTP: Qinghai-Tibet Plateau
SEOs: significantly expanded orthogroups
WGD: whole-genome duplication
LTR-retrogene: retrogene flanked by LTRs
LTR-gene: non-retrogenes flanked by LTRs.
