# Supplementary material for: Rapid Genome Evolution and Adaptation of Thlaspi arvense Mediated by Recurrent RNA-Based and Tandem Gene Duplications
Source: Front Plant Sci. 2022 Jan 4;12:772655. doi: 10.3389/fpls.2021.772655 (PMC8764390; doi:10.3389/fpls.2021.772655)
Supplement: Supplementary file 1 [file Data_Sheet_1.pdf]

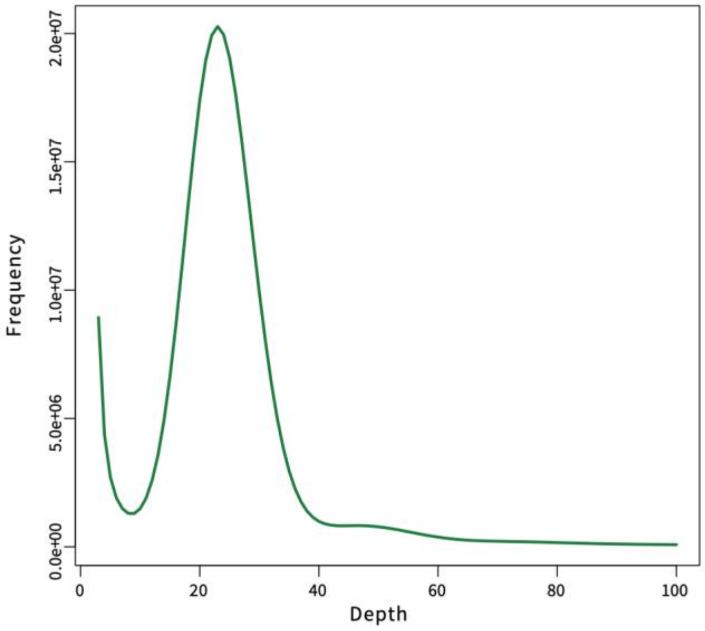

Figure S1 k-mer distribution of *T. arvense* genome using Illumina reads.

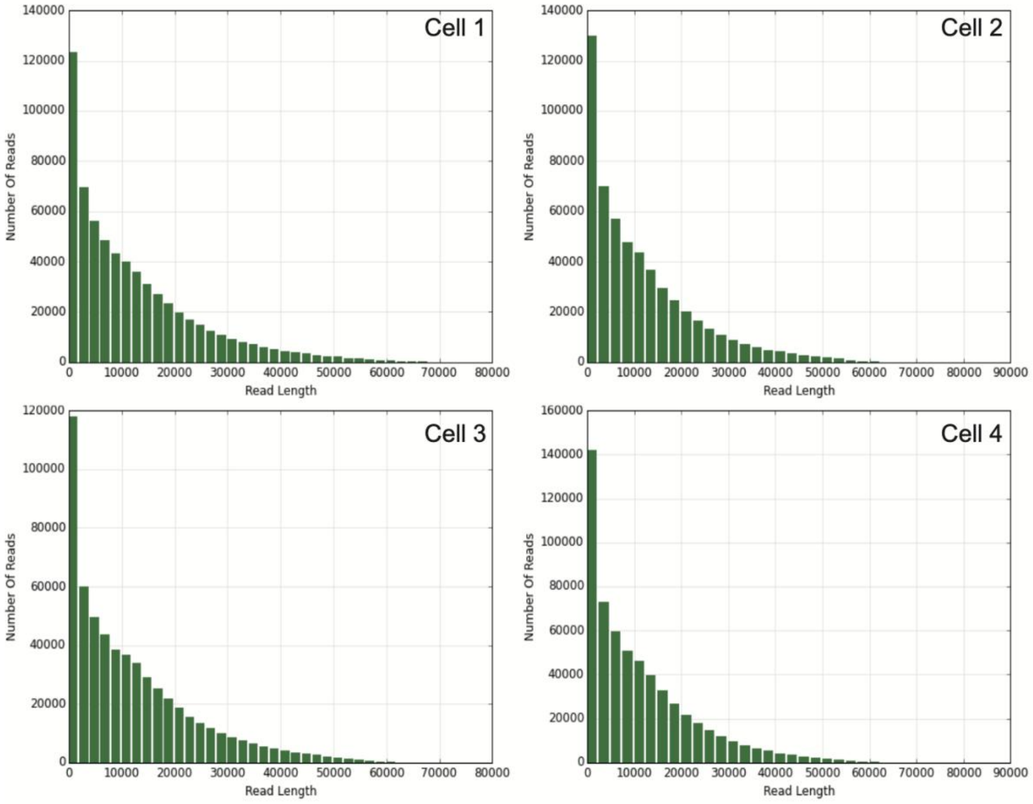

Figure S2 Length distribution of *T. arvense* genome SMRT sequencing reads from PacBio Sequel platform.

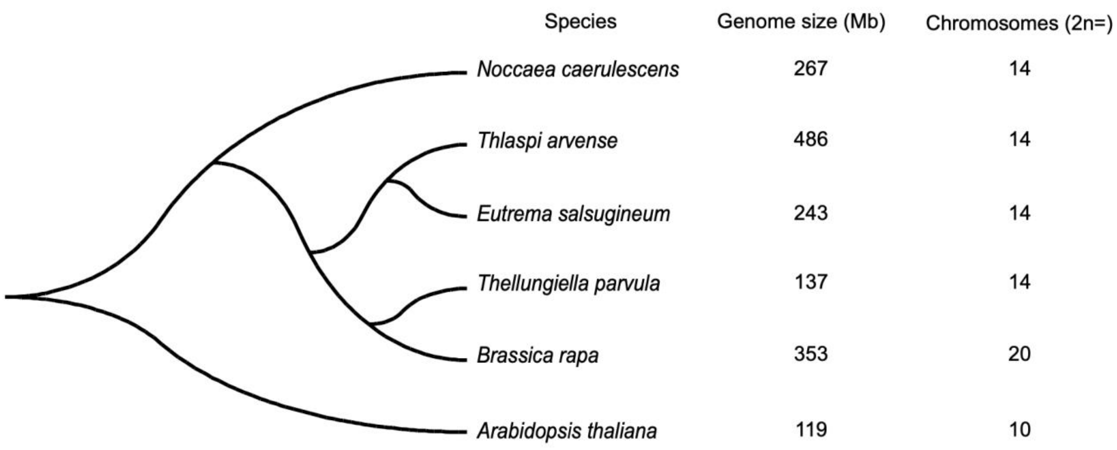

Figure S3 Genome sizes of close relatives of *T. arvense* in Brassicaceae. The phylogenetic relationship of these Brassicaceae species was cited from Huang et al. (2016).

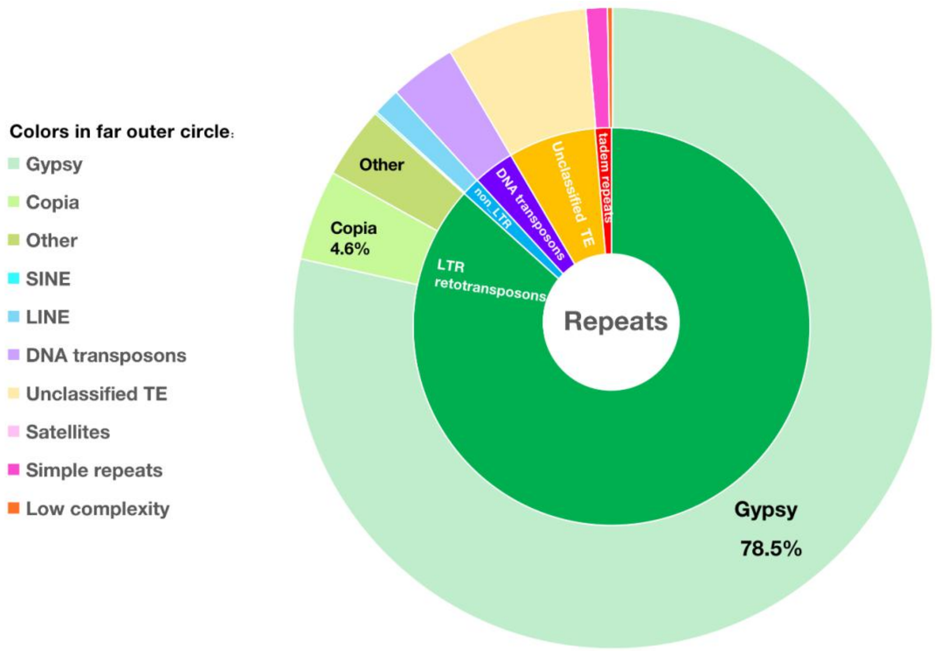

Figure S4 The propositions of different types of repeats in the *T. arvense* genome.

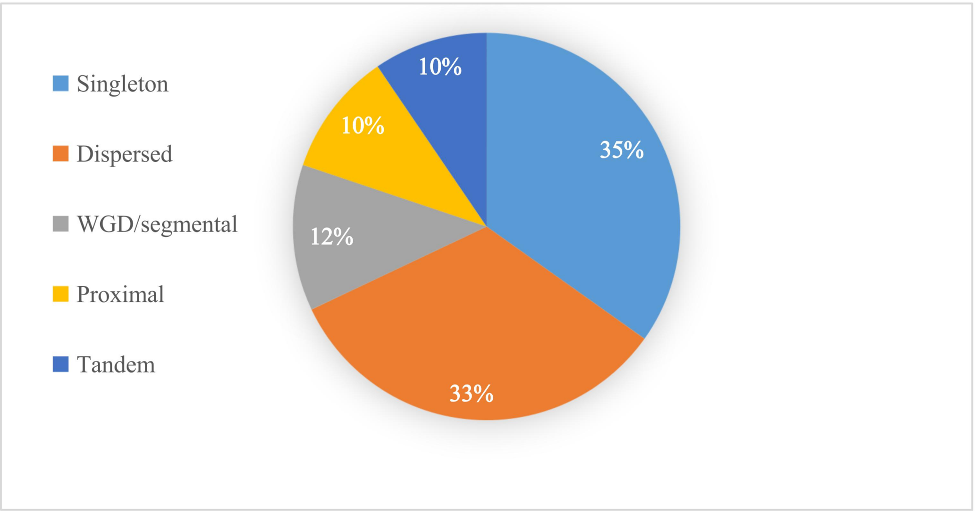

Figure S5 The propositions of different types of gene duplication in the *T. arvense* genome.

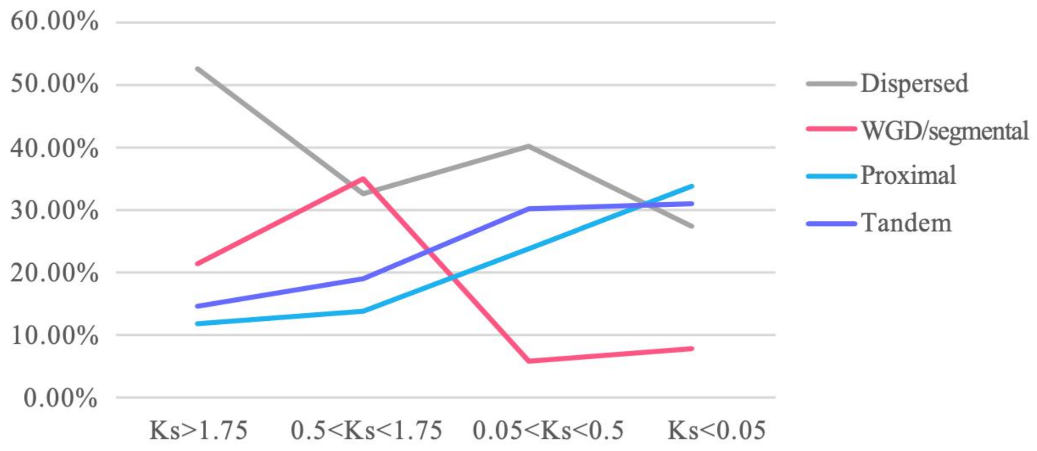

Figure S6 Changes of the percentage of types of duplicated genes in the *T. arvense* genome over time.

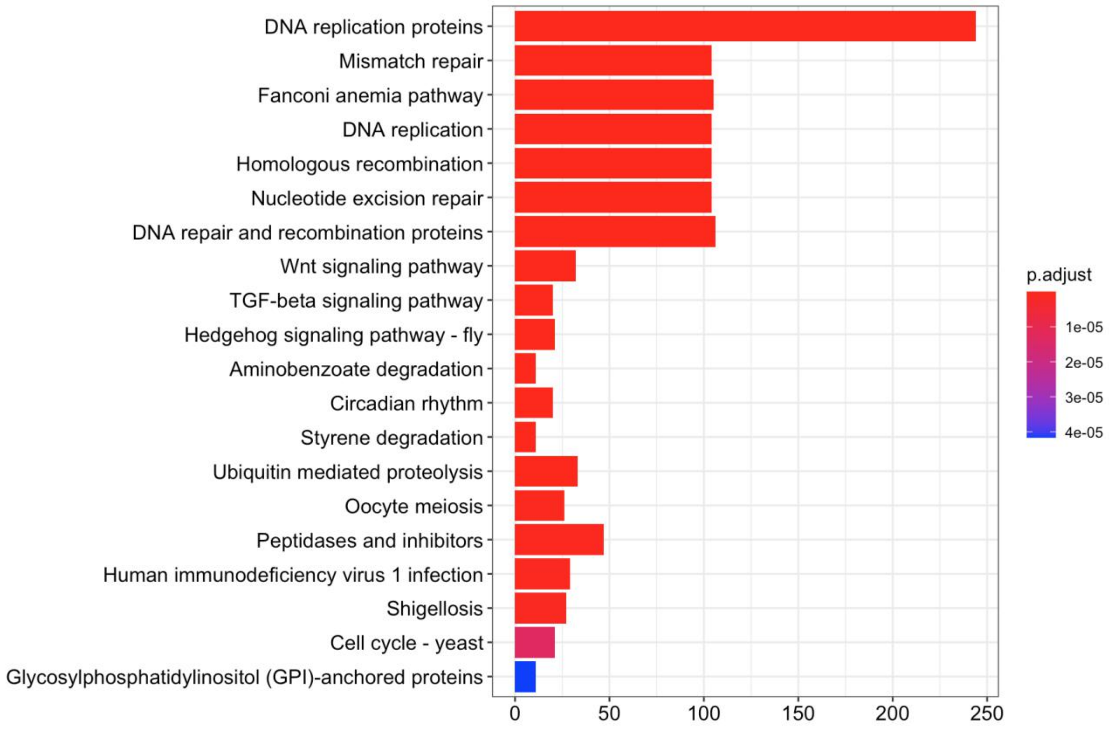

Figure S7 KEGG annotation enrichment of genes of significantly expanded orthogroups in the *T. arvense* genome.

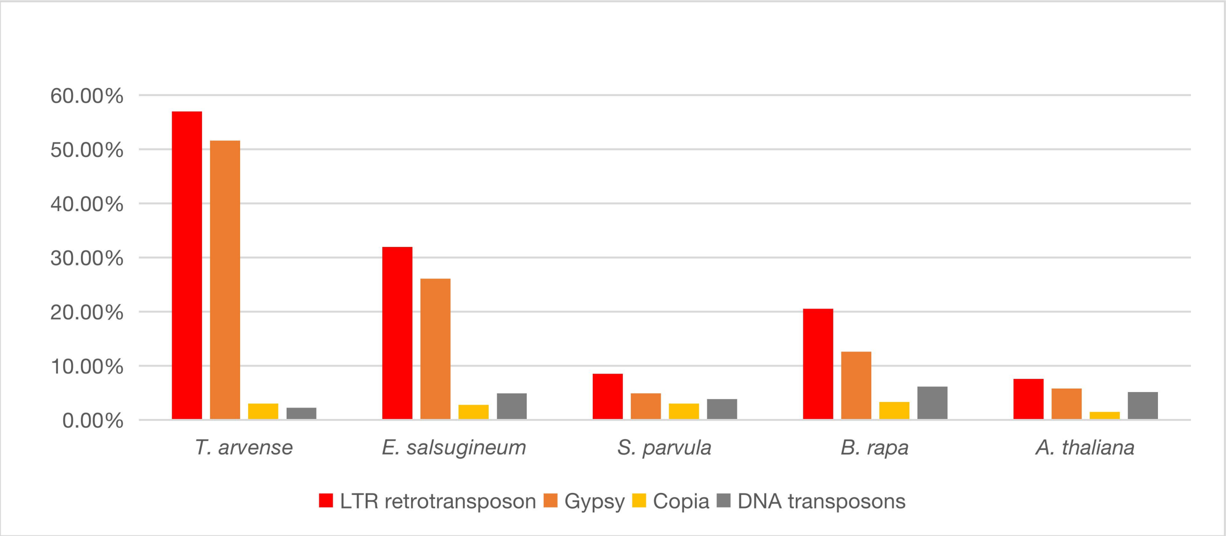

Figure S8 LTR retrotransposons and DNA transposons percentages of genomes assemblies of five Brassicaceae species.

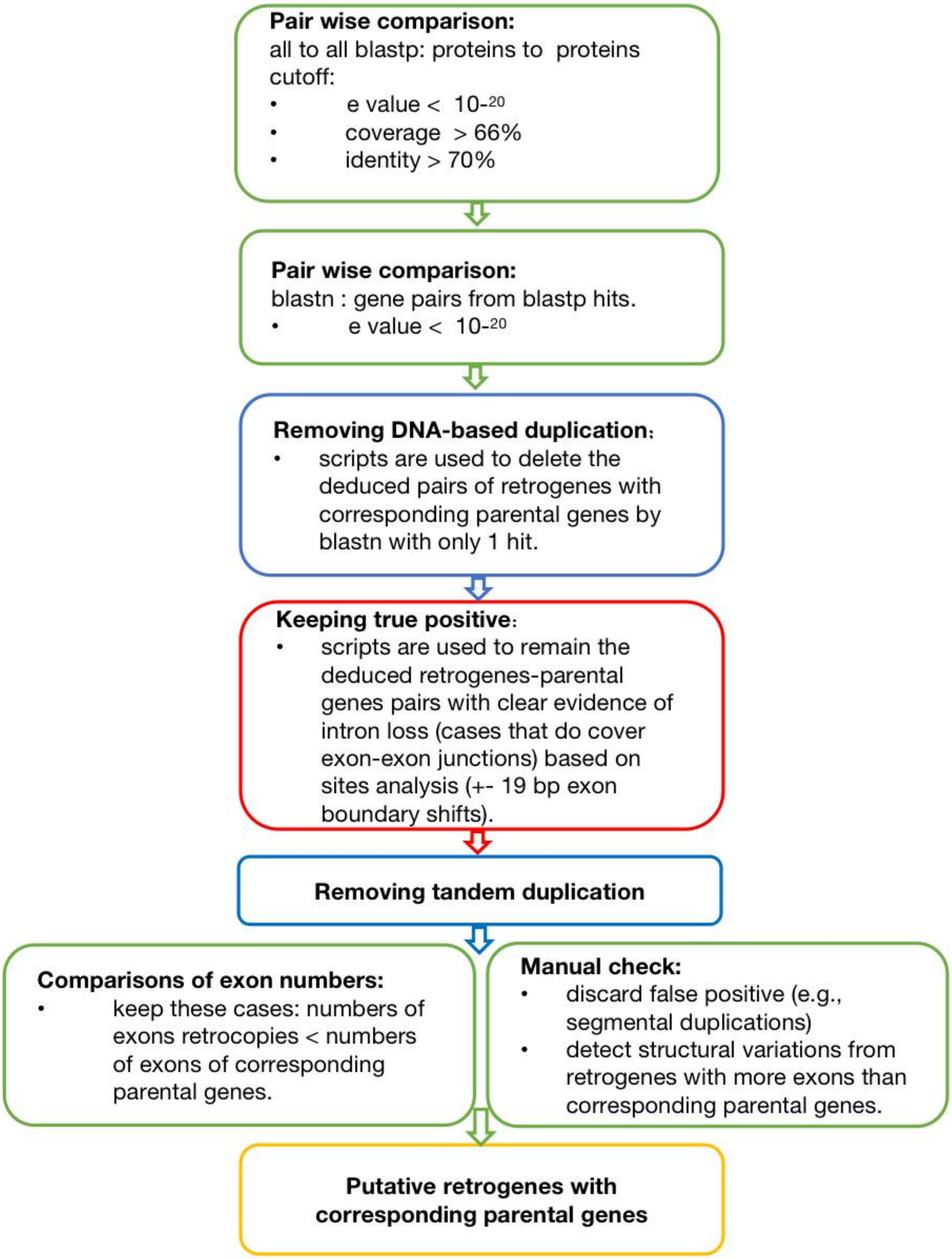

Figure S9 Flow of retrogenes identification in the *T. arvense* genome.

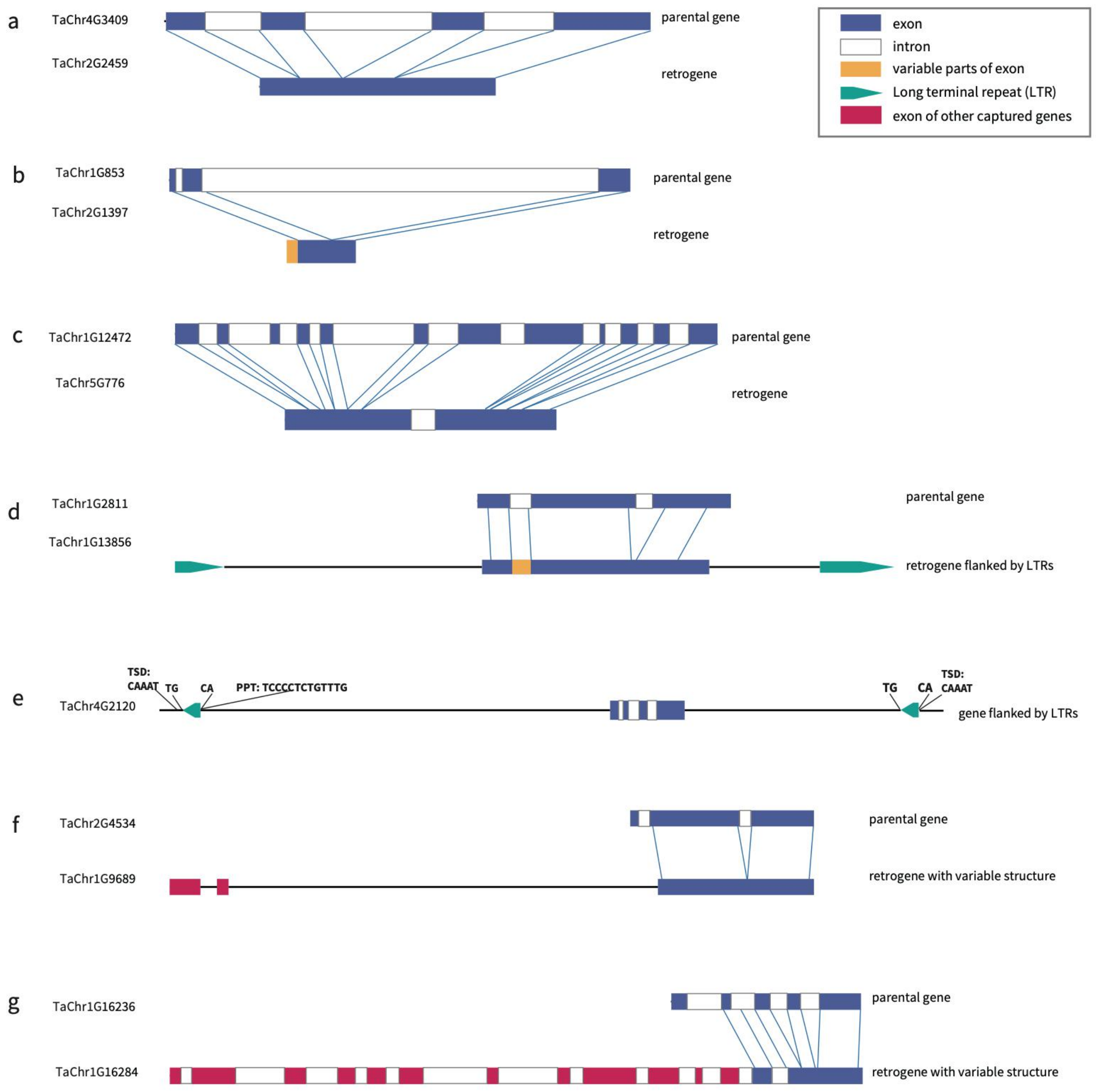

Figure S10 Models for the emergence of retrogenes containing single or multiple exons in the *T. arvense* genome. a, Classical retrogene with one exon; b, Retrogene emergence after intron retention (the first intron of the parental gene) and with new parts of exons adding; c, Retrogene formed by exon skipping (the third exon of the parental gene) and intron retention (the seventh intron of the parental gene); d, Retrogene flanked by LTRs with a variable part of the exon; e, A gene harboring Hsp70 domain flanked by LTRs; f, A retrogene capturing two exons of other genes with unknown domain; g, A retrogene captured by an ABC transporter gene.

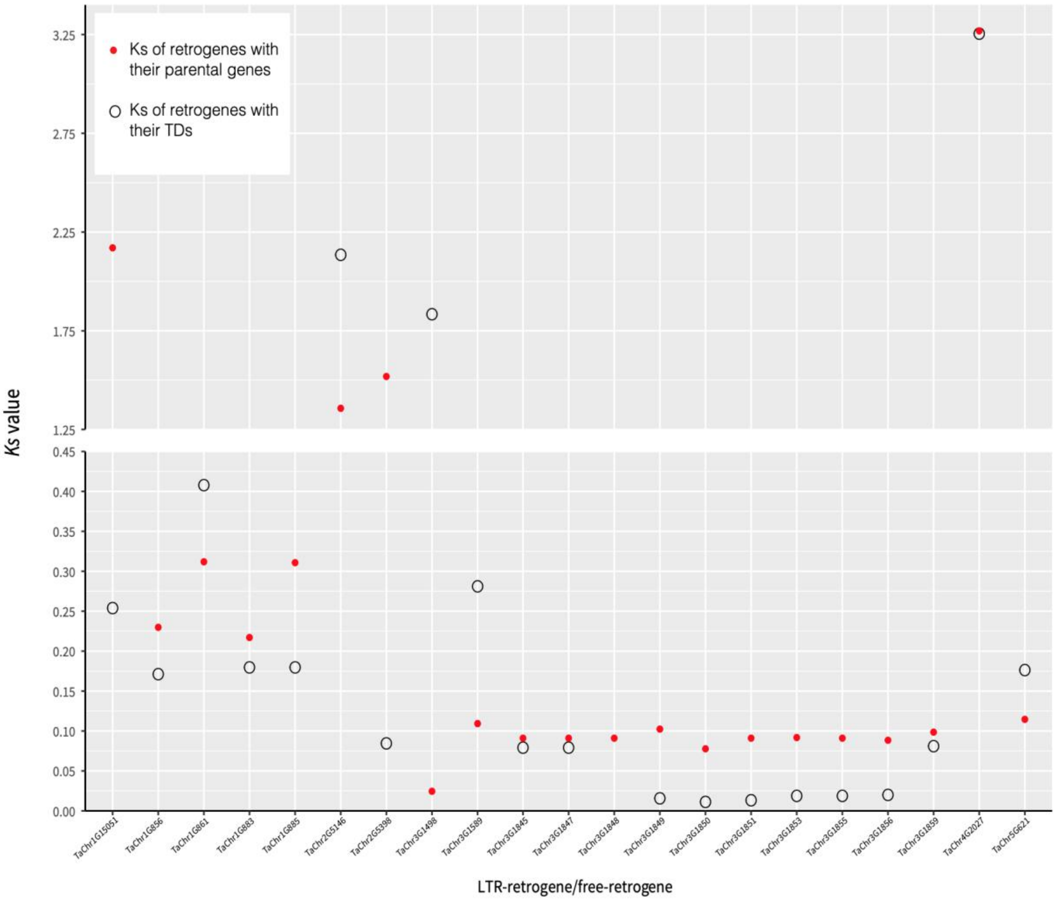

Figure S11 *Ks* values of LTR-retrogenes/free-retrogenes, which are TDs themselves with their parental genes and with their TDs in SEOs which possess TDs more than 40% in *T. arvense* genome. “TDs” indicates the tandem duplicates.

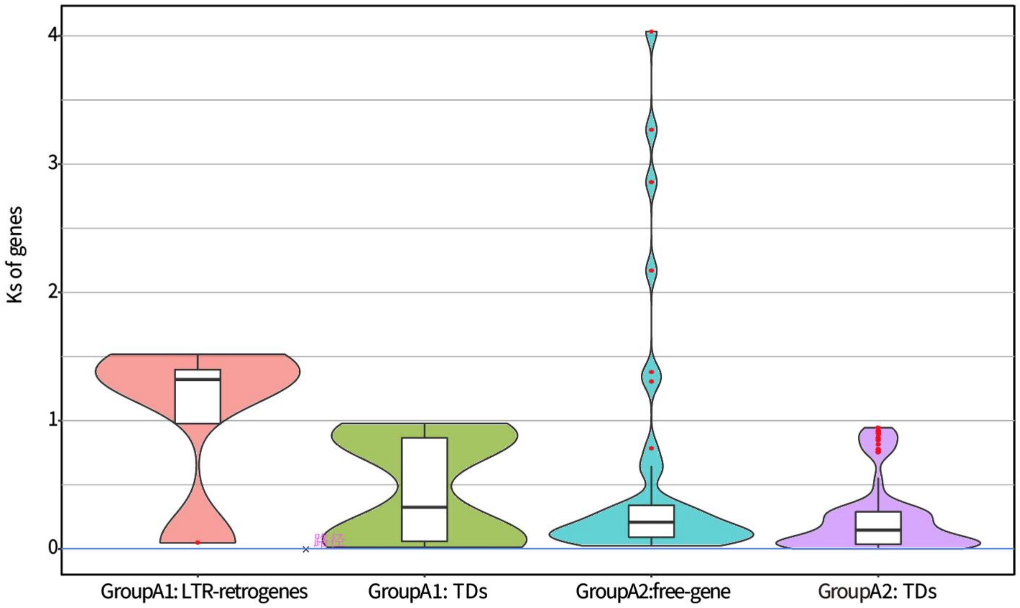

Figure S12 *Ks* values of LTR-retrogenes/free-retrogenes with relevant parental genes and *Ks* values of all TDs in Group A1 and A2 in the *T. arvense* genome. “TDs” indicates the tandem duplicates.

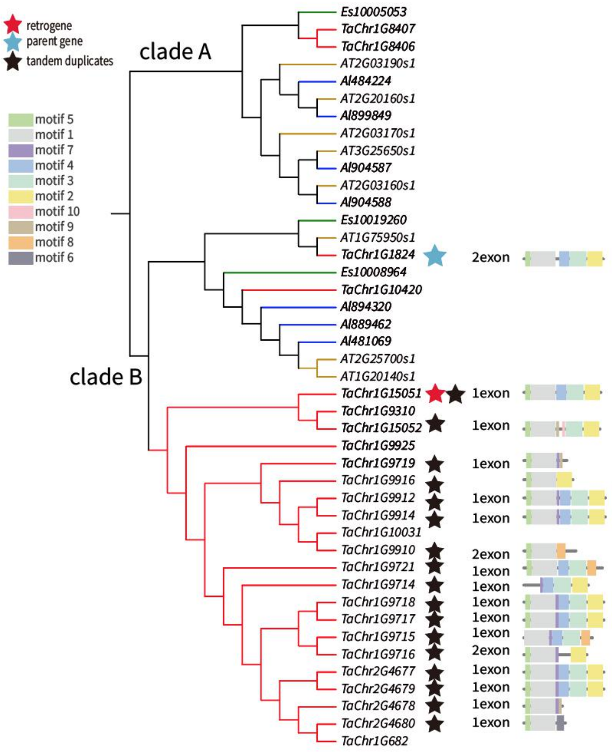

Figure S13 The phylogenetic tree and motifs of *SKP1* family in *T. arvense* (branches were colored in red), *E. salsugineum* (branches were colored in green), *A. lyrata* (branches were colored in blue), *and A. thaliana* (branches were colored in earth yellow).

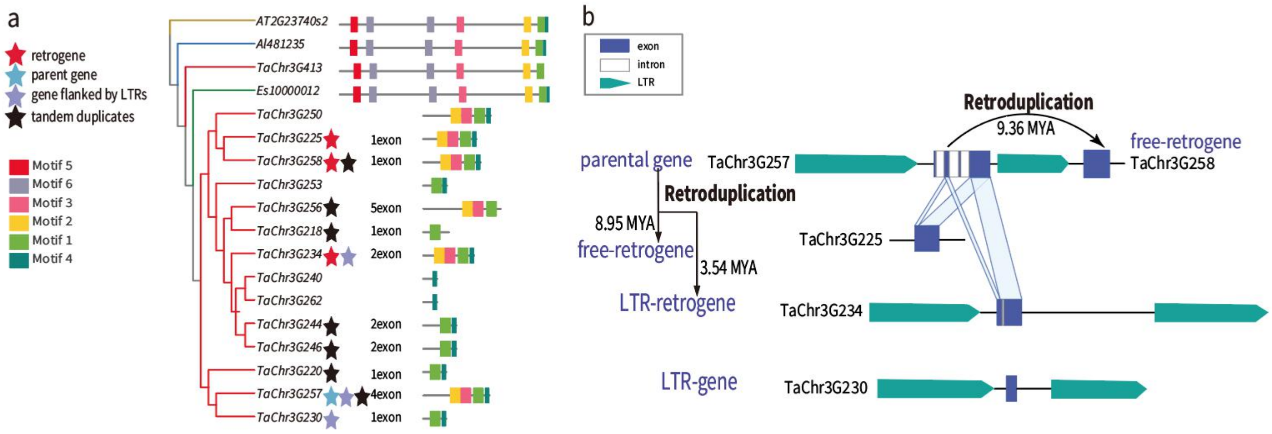

Figure S14 *SET* gene family. a, the phylogenetic tree and motifs of *SET* family in *T. arvense* (branches were colored in red), *E. salsugineum* (branches were colored in green), *A. lyrata* (branches were colored in blue), and *A. thaliana* (branches were colored in earth yellow); b, Models for the evolution of *SET* gene through retroduplication.

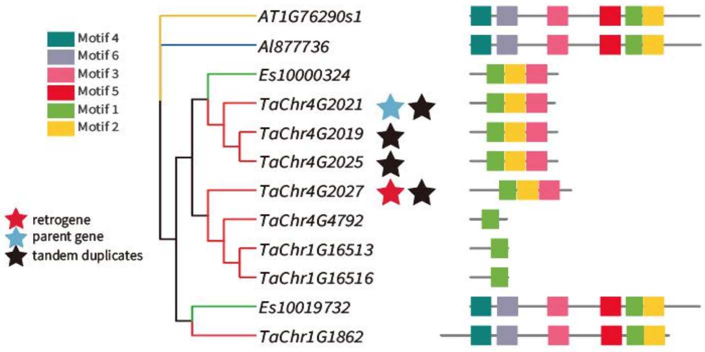

Figure S15 The phylogenetic tree and motifs of AMP-binding (*AMPB)* family in *T. arvense* (branches were colored in red), *E. salsugineum* (branches were colored in green), *A. lyrata* (branches were colored in blue), and *A. thaliana* (branches were colored in earth yellow).

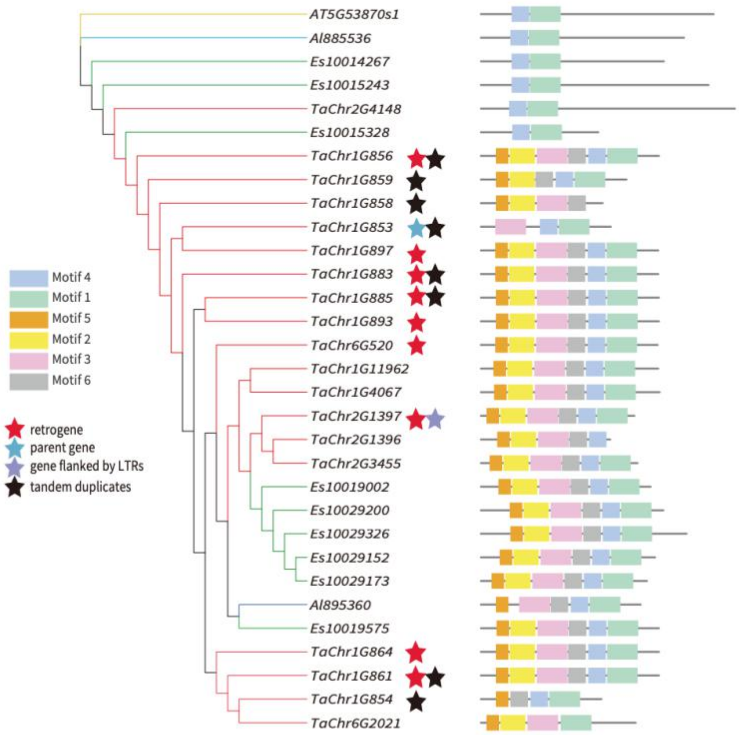

Figure S16 The phylogenetic tree and motifs of *Cu_bind_like* (*PCL*) gene family in *T. arvense* (branches were colored in red), *E. salsugineum* (branches were colored in green), *A. lyrata* (branches were colored in blue), and *A. thaliana* (branches were colored in earth yellow).

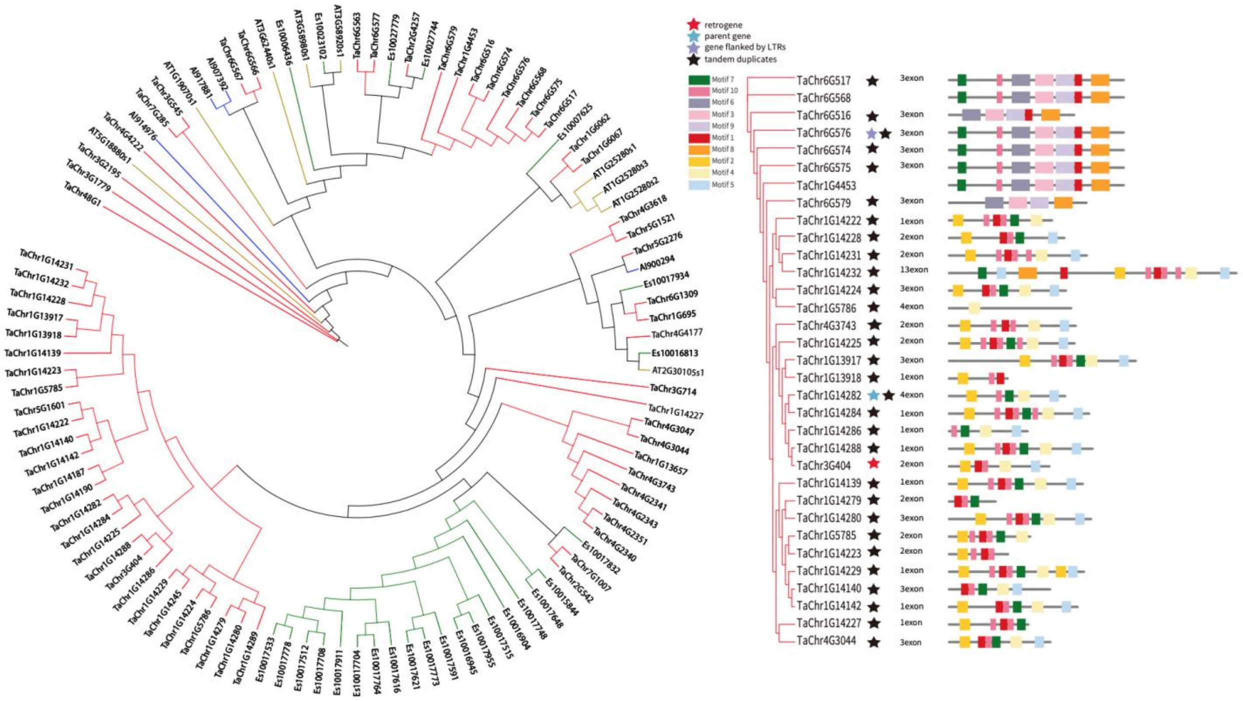

Figure S17 The phylogenetic tree and motifs of *F-box* and leucine-rich repeat (*F-box*) gene family in *T. arvense* (branches were colored in red), *E. salsugineum* (branches were colored in green), and *A. thaliana* (branches were colored in earth yellow).

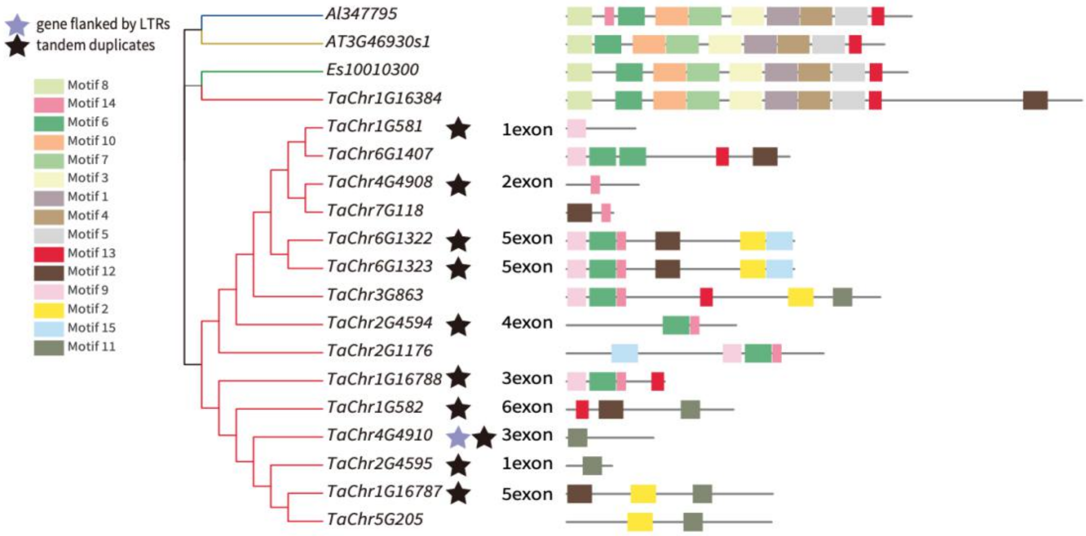

Figure S18 The phylogenetic tree and motifs of *Cullin* gene family in *T. arvense* (branches were colored in red), *E. salsugineum* (branches were colored in green), *A. lyrata* (branches were colored in blue), and *A. thaliana* (branches were colored in earth yellow).

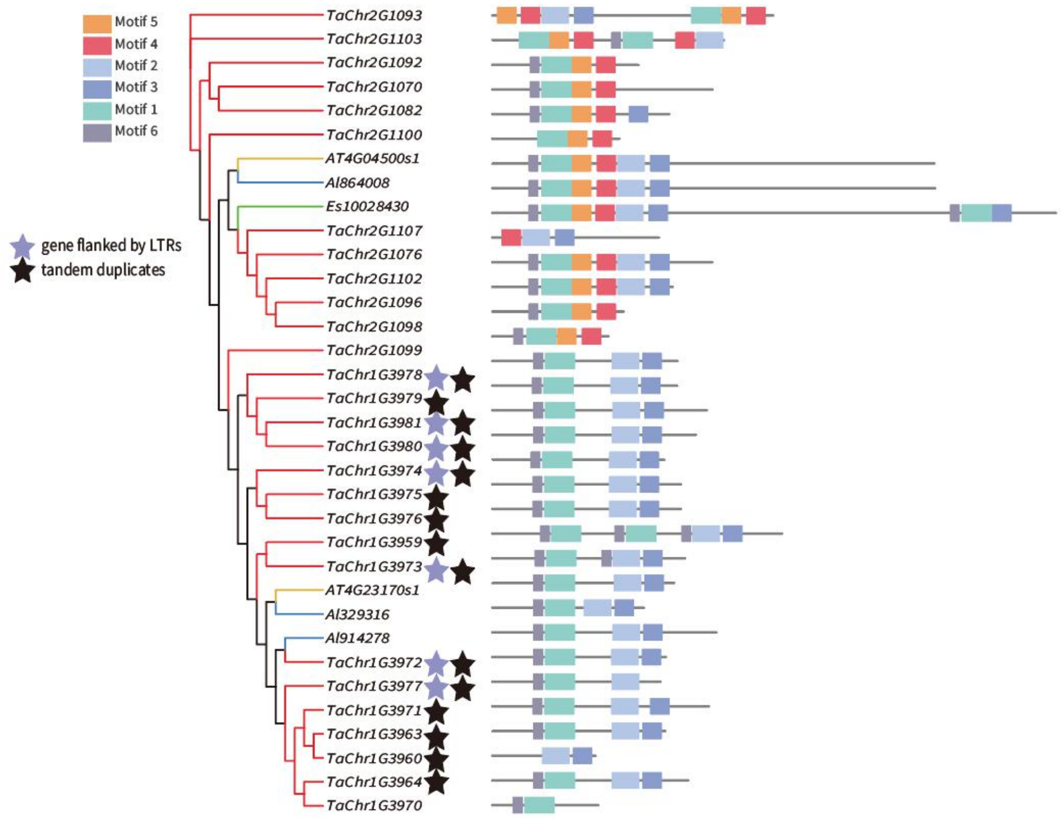

Figure S19 The phylogenetic tree and motifs of *Cysteine-rich receptor-like kinases* (*CRK*) gene family in *T. arvense* (branches were colored in red), *E. salsugineum* (branches were colored in green), *A. lyrata* (branches were colored in blue), and *A. thaliana* (branches were colored in earth yellow).

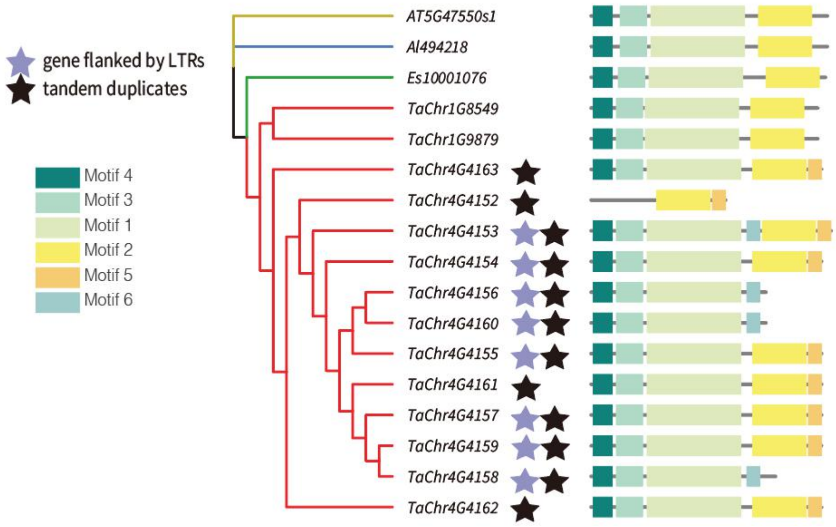

Figure S20 The phylogenetic tree and motifs of *Aspartic proteinase inhibitors* (*API*) gene family in *T. arvense* (branches were colored in red), *E. salsugineum* (branches were colored in green), *A. lyrata* (branches were colored in blue), and *A. thaliana* (branches were colored in earth yellow).

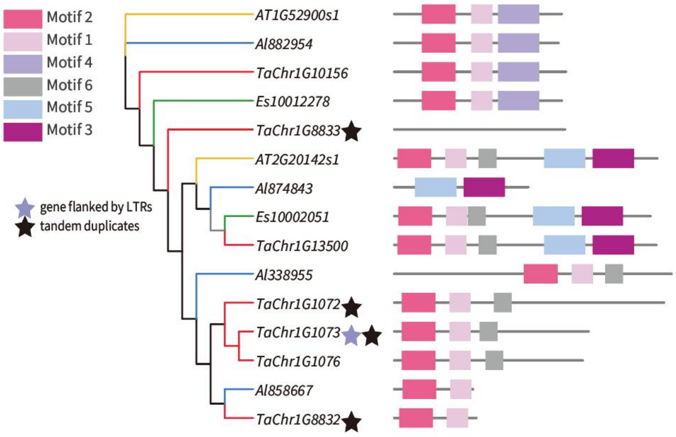

Figure S21 The phylogenetic tree and motifs of *TIR* gene family in *T. arvense* (branches were colored in red), *E. salsugineum* (branches were colored in green), *A. lyrata* (branches were colored in blue), and *A. thaliana* (branches were colored in earth yellow).

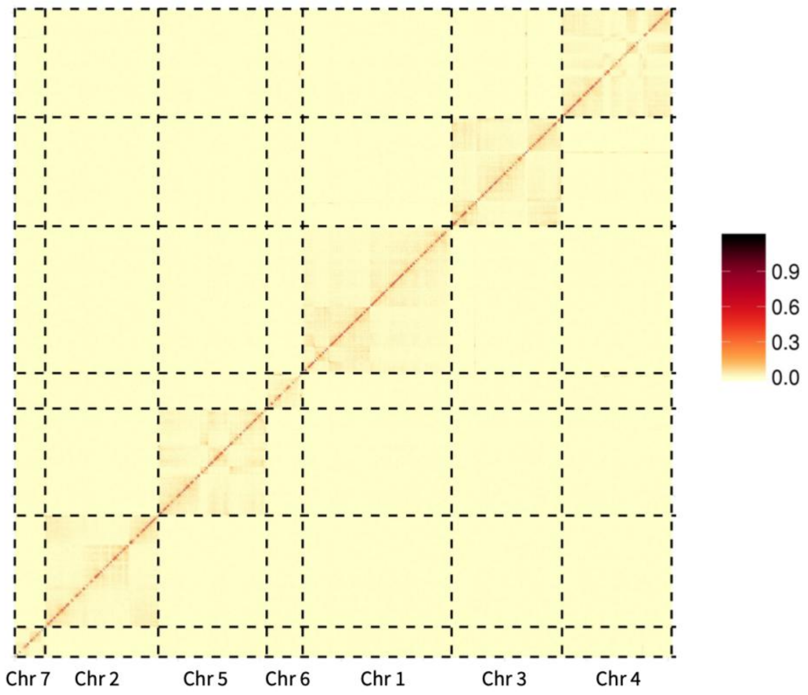

Figure S22 Hi-C contact maps for *T. arvense* genome. Validation of Hi-C-assisted pseudochromosome assembly by calculating the thermal interaction correlation.
